# Transcriptome analysis reveals absence of unintended effects in drought-tolerant transgenic plants overexpressing the transcription factor *ABF3*

**DOI:** 10.1186/1471-2164-11-69

**Published:** 2010-01-28

**Authors:** Ashraf Abdeen, Jaimie Schnell, Brian Miki

**Affiliations:** 1 Agriculture and Agri-Food Canada, 960 Carling Ave., Ottawa, ON, K1A 0C6 Canada; 2 Current address: Department of Biology, McGill University, 1205 Docteur Penfield Ave., Montreal, QC, H3A 1B1 Canada; 3 Current address: Plant and Biotechnology Risk Assessment Unit, Canadian Food Inspection Agency, 1400 Merivale Rd., Ottawa, ON, K1A 0Y9 Canada

## Abstract

**Background:**

Plants engineered for abiotic stress tolerance may soon be commercialized. The engineering of these plants typically involves the manipulation of complex multigene networks and may therefore have a greater potential to introduce pleiotropic effects than the simple monogenic traits that currently dominate the plant biotechnology market. While research on unintended effects in transgenic plant systems has been instrumental in demonstrating the substantial equivalence of many transgenic plant systems, it is essential that such analyses be extended to transgenic plants engineered for stress tolerance. Drought-tolerant *Arabidopsis thaliana *were engineered through overexpression of the transcription factor *ABF3 *in order to investigate unintended pleiotropic effects. In order to eliminate position effects, the Cre/*lox *recombination system was used to create control plant lines that contain identical T-DNA insertion sites but with the *ABF3 *transgene excised. This additionally allowed us to determine if Cre recombinase can cause unintended effects that impact the transcriptome.

**Results:**

Microarray analysis of control plant lines that underwent Cre-mediated excision of the *ABF3 *transgene revealed only two genes that were differentially expressed in more than one plant line, suggesting that the impact of Cre recombinase on the transcriptome was minimal. In the absence of drought stress, overexpression of *ABF3 *had no effect on the transcriptome, but following drought stress, differences were observed in the gene expression patterns of plants overexpressing *ABF3 *relative to control plants. Examination of the functional distribution of the differentially expressed genes revealed strong similarity indicating that unintended pathways were not activated.

**Conclusions:**

The action of ABF3 is tightly controlled in *Arabidopsis*. In the absence of drought stress, ectopic activation of drought response pathways does not occur. In response to drought stress, overexpression of *ABF3 *results in a reprogramming of the drought response, which is characterized by changes in the timing or strength of expression of some drought response genes, without activating any unexpected gene networks. These results illustrate that important gene networks are highly regulated in *Arabidopsis *and that engineering stress tolerance may not necessarily cause extensive changes to the transcriptome.

## Background

Drought is a major abiotic stress that limits crop productivity [1]. Climate change models predict an increase in summer drying in the midlatitudes, which could contribute to an increase in the number of episodes of drought [2,3]. Engineering plants with enhanced tolerance of abiotic stresses such as drought is a major objective of plant biotechnology that is expected to be commercialized in the near future [4,5]. Tolerance to abiotic stress may be achieved through the modification of endogenous plant pathways, often by manipulating important regulatory proteins such as transcription factors. Altering the level of expression of key transcription factors involved in abiotic stress pathways has been shown to enhance tolerance to various abiotic stresses in Arabidopsis [6-9] as well as in important crop species such as rice [10-12], maize [13], and alfalfa [14].

Traits involving tolerance to abiotic stresses are considered to be more complex than those that are currently commercialized due to the large number of genes and pathways that may be affected. Furthermore, the interaction between plants and the environment is an intricate, continuous process that has been difficult to characterize, further adding to the complexity of manipulating abiotic stress tolerance traits. The increased complexity of these traits may correspond with a greater potential for unintended effects to occur in transgenic plants.

In transgenic systems, two different types of unintended effects are generally known to occur [15]. Position effects are attributed to the insertion of a transgene at a particular locus in the genome and the resulting interference this might cause. These effects will vary with the site of integration and will therefore be unique to each independent plant line. Position effects can be easily eliminated by screening for plant lines that have no or little position effects. In contrast, pleiotropic effects are independent of the site of transgene insertion and are the sum of all the phenotypic effects caused by expression of the transgene. While some of these may be the intended trait, others may occur through unexpected interactions of the gene with plant processes and constitute the unintended pleiotropic effects. These effects are of greater interest since they are more difficult to eliminate and more likely to create safety issues.

Engineering more complex traits such as abiotic stress tolerance in plants through the manipulation of transcription factors may uncover cryptic properties of the transcription factor that could produce some of the unintended pleiotropic effects. Many transcription factors are part of large families that have complex evolutionary histories [16,17]. These families typically arise through gene duplications followed by functional divergence in separate expression domains or through the acquisition of new functions. These processes often result in functional redundancies within the families that can be difficult to detect. Furthermore, some transcription factors may retain ancestral functions that are sometimes only revealed by altering the normal pattern of expression. Therefore the manipulation of transcription factors in engineering complex traits such as abiotic stress tolerance may be likely to produce unintended pleiotropic effects.

The use of non-targeted global profiling technologies, such as microarray analysis, to identify unintended effects in plant systems has proven an effective means of determining the "substantial equivalence" of a transgenic plant to its non-transgenic counterpart. Such approaches have been used to investigate unintended effects in a number of transgenic plant systems [18-27]. To date, these studies have primarily focused on simple, monogenic traits such as those that are currently commercially grown. As transgenic crops with more complex traits involving the modification of endogenous plant pathways will soon be entering the market, it is important to extend these analyses to investigate the potential for unintended pleiotropic effects in such systems.

In order to understand the extent and kinds of unintended effects that could be induced in transgenic plants engineered for complex traits, we conferred drought tolerance on *Arabidopsis thaliana *by overexpressing the transcription factor *ABF3*. This system targets drought resistance, a trait that will likely enter the market in the near future. Since transcription factors ultimately function by altering the levels of expression of target genes, we investigated unintended effects using microarray analysis to survey global gene expression profiles. In order to eliminate position effects in our analysis and focus on the pleiotropic unintended effects, we employed the Cre/l*ox *system to excise the *ABF3 *transgene from the site of insertion, leaving behind the selectable marker, to create control plant lines. Without the ABF3 transgene, the pleiotropic effects will be absent but the site of integration is still interrupted by the selectable marker such that position effects are maintained in these lines.

ABF3 belongs to the ABF/AREB subfamily of bZIP transcription factors which consists of thirteen members in *Arabidopsis*. Several members have been shown to function in ABA signalling either during seed maturation or in response to stress [28]. These factors can bind to ABA-response elements (ABREs), *cis*-regulatory elements found in the promoters of many ABA- and stress-responsive genes [29-31]. In addition to drought tolerance, overexpression of *ABF3 *confers tolerance to salt, cold, heat, and oxidative stresses, suggesting that it regulates multiple abiotic stress pathways in *Arabidopsis *[7,32]. Three other ABF/AREB transcription factors are predicted to function in ABA-dependent stress signalling based on expression profiling and overexpression studies. Expression of *ABF1 *is induced by cold-treatment [29]. *ABF2/AREB1 *is induced by salt-treatment as well as dehydration but not cold and overexpression confers tolerance to a wide range of abiotic stresses, including salt, drought, heat, and oxidative stress [29,31,33]. Interestingly, *ABF2/AREB1 *also appears to function in glucose signalling as well as in the regulation of seedling growth [33]. *ABF4/AREB2 *is expressed in response to cold, drought, and salt and overexpression renders plants tolerant to drought and salt [7,29,31]. Therefore, ABF3 likely shares some redundant functions with other members of the ABF/AREB subfamily.

Plant response to drought involves changes in the expression patterns of a large number of genes [34-36] and, in addition to members of the ABF/AREB family, a number of other transcription factors have been identified that play a role in the drought response in *Arabidopsis*. These include the AP2/ERF transcription factors DREB2A, DREB2B, and CBF4 [8,37,38], AtMYB2, which functions in concert with AtMYC2 [39-41], and the NAC family transcription factors ANAC19, ANAC055/ATNAC3, and ANAC072/RD26 [42], which at least partially function in concert with a zinc finger homeodomain protein ZFHD1 [43]. Therefore, while overexpression of ABF3 affects one of the key drought response pathways, it is not the only pathway mediating the drought response at the gene expression level.

The use of the Cre/*lox *system to create control lines also created an opportunity to examine the effects of the Cre/l*ox *system on the transcriptome. Site-specific recombination technologies can be used to excise selectable markers or other undesirable genetic elements and can also be used to direct site-specific integration of transgenes [44]. While many studies have employed Cre-mediated recombination in plant systems with no apparent unintended effects [45-48], other studies have observed a range of abnormal phenotypes including growth defects, leaf chlorosis, delayed flowering, and male sterility [49,50]. In tobacco plants transformed with a chloroplast targeted Cre recombinase, recombination was observed involving cryptic *lox *sites in the plastid genome, but invariably the second lox site was located within the transgene [51-53]. These recombinations could result in deletions of up to 147 kb, but they did not cause any deleterious effects in the plants [51-53]. Studies in animal systems have similarly revealed that Cre recombinase can have unintended effects, often leading to chromosomal aberrations [54-57].

These studies suggest that the Cre/*lox *system has the potential to cause unintended effects in plant systems, by mediating recombination with cryptic *lox *sites that may be present in the genome resulting in large deletions. Such cryptic *lox *sites are difficult to identify since they may deviate substantially from conventional *loxP *sites [52] and not all of the unintended effects may produce readily apparent phenotypic abnormalities, so studying the unintended effects of Cre recombinase using a non-targeted approach such as microarray analysis is essential for establishing the utility and safety of this technology.

In this study, we performed microarray analysis on *Arabidopsis *plants engineered to be drought-tolerant through overexpression of the transcription factor *ABF3 *with the goal of identifying unintended pleiotropic effects. The results suggest that overexpression of *ABF3 *has a minimal impact on the transcriptome, with differences in the gene expression pattern only detectable in response to drought and then being suggestive of transcriptional reprogramming as opposed to the activation of novel pathways. In addition, we examined the impact of Cre recombinase on the transcriptome to detect any unintended effects of this technology and found that it had minimal effects on gene expression patterns in plants following transgene excision.

## Results

### Phenotype of plants overexpressing ABF3

**Figure 1:**
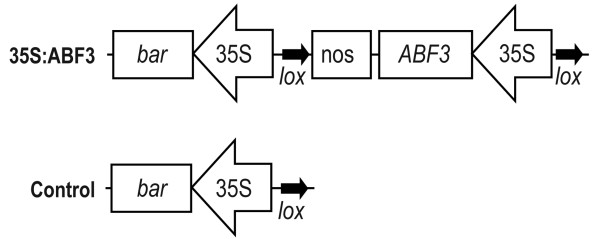
**35S:ABF3 and control T-DNA regions**. Schematic map of the 35S:ABF3 and control T-DNA regions. *ABF3*, coding region of *ABF3 *gene; 35S, CaMV 35S promoter; nos, *nopaline synthase *transcriptional terminator; *bar*, bialaphos resistance; *lox*, *loxP *sites.

A construct was created containing the CaMV 35S promoter followed by the coding sequence of *ABF3 *and then the *nopaline synthase *transcriptional terminator, all of which was flanked by two *loxP *sites (Figure 1A). This construct was transformed into *Arabidopsis thaliana *to generate 35S:ABF3 plants. Fifty-nine independent transformants were recovered and three high-expressing lines containing single insertions were selected for further analysis (35S:ABF3-48, -57, and -59).

Control plant lines were generated by excising the 35S:ABF3 transgene, leaving only the selectable marker transgene at the site of insertion (Figure 1B). This was achieved by crossing 35S:ABF3 plants with plants expressing the *Cre *recombinase gene and then backcrossing the progeny to wild-type *Arabidopsis *to remove the *Cre *recombinase gene, generating three control lines (Control-48, -57, and -59). The loss of both the *ABF3 *transgene and the *Cre *recombinase transgene was confirmed by PCR (Additional file 1).

**Figure 2:**
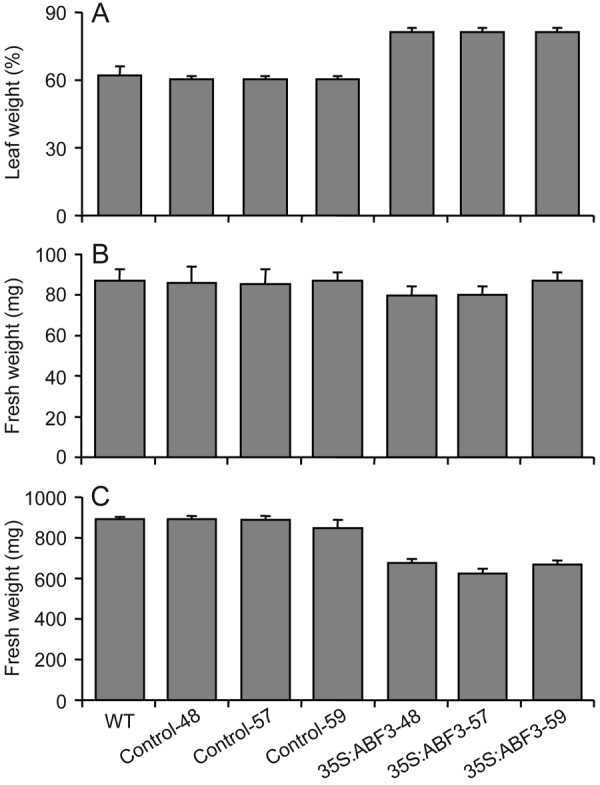
**Phenotype of 35S:ABF3 plants**. (A) Transpiration rate of leaves from 35S:ABF3, control, and wild-type plants. Leaves from 4-week-old plants were excised and left at room temperature for one day and the percentage of leaf weight remaining was measured (n = 5). For each measurement, five leaves were pooled. Error bars indicate standard deviation. (B, C) Growth retardation of 35S:ABF3 plants. Three-day-old seedlings were transplanted to fresh MS plates and fresh weight was measured after 1 (B) (n = 6) or 4 weeks (C) (n = 5) of growth. For each measurement, eight (B) or 5 (C) seedlings were pooled. Error bars indicate standard deviation.

Previously, *Arabidopsis *plants overexpressing *ABF3 *were found to be more tolerant to drought conditions, which could at least partially be attributed to a lower rate of transpiration [7]. To confirm that the 35S:ABF3 lines show a similar phenotype, the transpiration rate was determined. Excised leaves from 4-week-old 35S:ABF3 plants left at room temperature for 1 day lost less fresh weight than both wild-type and control plants, indicating a lower rate of transpiration (Figure 2A). In addition, 35S:ABF3 plants showed a mild growth retardation that became more evident as plants matured (Figure 2B, C), which is also consistent with previous results [7].

### Identification of position effects and impact of Cre recombinase on the transcriptome

Microarray analysis of control plant lines was performed to identify position effects as well as to determine if use of the Cre/*lox *system can cause unintended effects in plant systems. Position effects should be specific to each of the three control lines, as each line is expected to have a unique insertion site. In contrast, unintended effects resulting from excision at cryptic *lox *sites are more likely to affect independent plant lines similarly, since cryptic sites that may be present in the *Arabidopsis *genome will equally be found in each plant line.

Using a *P*-value cut-off of 0.05, only a small number of genes were differentially expressed in control lines compared to wild-type plants (Table 1). In the Control-48 line, only a single gene was differentially expressed. In the Control-57 and -59 lines, 10 and 4 genes were differentially expressed and two of these, *rps7 *(AtCg00900/AtCg01240) and *rps12.1 *(transplice part 1 of 2) (AtCg00065), were common to both lines. These genes could represent either position effects or natural background variation in gene expression. The *rps7 *and *rps12.1 *genes that were differentially expressed in both Control-57 and -59 lines may reflect unintended effects of the Cre/*lox *system.

**Table 1:** Genes differentially expressed (*P *< 0.05) in control lines following Cre-mediated excision of the *ABF3 *transgene

Line	Probe Set ID	AGI	Annotation	M	P
**48**	267442_at	At2g19080	Metaxin-related	0.68	1.22 × 10^-2^

**57**	244939_at	AtCg00065	Ribosomal protein s12 (Transplice part 1 of 2) (rps12.1)*	2.58	9.11 × 10^-4^
	244992_s_at	AtCg00900/AtCg01240	Ribosomal protein s7 (rps7)*	1.50	2.97 × 10^-2^
	245608_at	At4g14350	Protein kinase family protein	-0.99	5.86 × 10^-3^
	247137_at	At5g66210	Calcium-dependent protein kinase family protein (CDPK28)	-1.20	3.35 × 10^-2^
	247754_at	At5g59080	Expressed protein	1.33	2.20 × 10^-2^
	248812_at	At5g47330	Palmitoyl protein thioesterase family protein	2.34	7.24 × 10^-3^
	252053_at	At3g52400	Syntaxin, putative (SYP122)	-1.09	3.60 × 10^-2^
	255655_at	At4g00980	Zinc knuckle (CCHC-type) family protein	1.39	4.60 × 10^-6^
	259072_at	At3g11700	Fasciclin-like arabinogalactin protein 18 precursor (FLA18)	-0.73	2.29 × 10^-3^
	259348_at	At3g03770	Leucine-rich repeat transmembrane protein kinase, putative	0.91	1.82 × 10^-2^

**59**	244939_at	AtCg00065	Ribosomal protein s12 (Transplice part 1 of 2) (rps12.1)*	2.50	1.52 × 10^-3^
	244992_s_at	AtCg00900/AtCg01240	Ribosomal protein s7 (rps7)*	1.51	2.69 × 10^-2^
	246727_at	At5g28010	Bet v I allergen family protein	2.02	1.10 × 10^-5^
	262719_at	At1g43590	Transposable element gene	1.29	1.30 × 10^-2^

M is log2 fold change and P is FDR-adjusted *P*-value for Student's *t*-test.

* indicates genes encoded by the chloroplast genome.

### Effect of ABF3 overexpression on the transcriptome in the absence of drought

Microarray analysis of 35S:ABF3 plants was performed to identify unintended effects resulting from overexpression of a transcription factor in the absence of stress. Expression of *ABF3 *in the absence of stress is generally very low but it is rapidly induced in response to ABA [7,29]. Overexpression of *ABF3 *would therefore be expected to initiate those pathways that are typically activated in response to stress via ABA-mediated signalling. This has been observed for other transcription factors and is generally used as a means of identifying targets of that particular transcription factor [39,42,58].

Three independent plant lines containing the 35S:ABF3 transgene were compared to the corresponding control plant lines from which the 35S:ABF3 transgene was excised. Based on microarray analysis, using a cut-off of *P *< 0.05, only a small number of genes (7, 1, and 8) were differentially expressed in the three 35S:ABF3 transgenic plant lines (Table 2). The only gene that was differentially expressed in all three plant lines was *ABF3*.

**Table 2:** Genes differentially expressed (*P *< 0.05) in 35S:ABF3 transgenic plants

Line	Probe Set ID	AGI	Annotation	M	P
**48**	244964_at	AtCg00580	PSII cytochrome b559 (psbE)*	0.73	1.18 × 10^-2^
	245608_at	At4g14350	Protein kinase family protein	-1.54	1.27 × 10^-6^
	253263_at	At4g34000	ABA-responsive elements-binding factor 3 (ABF3)	3.35	2.09 × 10^-11^
	253399_at	At4g32850	Nuclear poly(A) polymerase (nPAP)	0.7	2.22 × 10^-2^
	256683_at	At3g52220	Expressed protein	-0.47	4.43 × 10^-2^
	256940_at	At3g30720	Expressed protein	1.68	3.25 × 10^-2^
	262719_at	At1g43590	Transposable element gene	2.02	3.12 × 10^-6^

**57**	253263_at	At4g34000	ABA-responsive elements-binding factor 3 (ABF3)	4.44	2.58 × 10^-14^

**59**	244992_s_at	AtCg00900/AtCg01240	Ribosomal protein s7 (rps7)*	-1.68	4.80 × 10^-3^
	244996_at	AtCg00160	Ribosomal protein s2 (rps2)*	-1.28	9.05 × 10^-3^
	245018_at	AtCg00520	Protein required for photosystem I assembly and stability (ycf4)*	-1.22	9.22 × 10^-3^
	245019_at	AtCg00530	Hypothethical protein (ycf10/cemA)*	-1.34	1.48 × 10^-2^
	245020_at	AtCg00540	Cytochrome f apoprotein (petA)*	-1.21	1.25 × 10^-2^
	253263_at	At4g34000	ABA-responsive elements-binding factor 3 (ABF3)	2.04	1.35 × 10^-6^
	261888_at	At1g80800	Pseudogene, 40S ribosomal protein S12 (rps12B)	-0.92	2.31 × 10^-2^
	264683_at	At1g65580	Inositol or phosphatidylinositol phosphatase/FRAGILE FIBRE 3 (FRA3)	-0.71	2.34 × 10^-2^

M is log2 fold change and P is FDR-adjusted *P*-value for Student's *t*-test.

* indicates genes encoded by the chloroplast genome.

### Drought response of 35S:ABF3 plants

Overexpression of *ABF3 *in *Arabidopsis *confers enhanced drought tolerance [7]. Since ABF3 is a transcription factor, it is likely that this is achieved at the level of gene expression. Therefore, microarray analysis should provide insight into the mechanism of drought tolerance. Since the expression pattern of *ABF3 *during the drought response is achieved by constitutive overexpression from the CaMV 35S promoter, it is possible that unintended effects may also be generated downstream. By comparing the transcriptional profile of 35S:ABF3 plants to control plants, it may be possible to identify these unintended effects. Two time points were examined in order to consider both early and late responses to drought. For this analysis, the Control-48 and 35S:ABF3-48 lines were selected as the Control-48 lines showed the smallest number of differentially expressed genes (Table 1).

Following 2 h of drought stress, using a cut-off of *P *< 0.05 and a fold-change of 2, 508 genes were differentially expressed in the control plants exposed to drought stress compared to unstressed control plants and 468 genes were differentially expressed in 35S:ABF3 plants exposed to drought stress compared to unstressed 35S:ABF3 plants. Following 24 h of drought stress, 592 genes were differentially expressed in control plants exposed to drought stress compared to unstressed control plants and 797 genes were differentially expressed in 35S:ABF3 plants exposed to drought stress compared to unstressed 35S:ABF3 plants.

In order to compare the profile of genes expressed in the two plant lines in response to drought stress, functional categorizations of the differentially expressed genes were obtained from The Arabidopsis Information Resource (TAIR; http://www.arabidopsis.org) and compared. Although the profile of genes differentially expressed in control and 35S:ABF3 plants show some differences, overall they show a similar pattern of distribution among the different functional classes (Additional file 2). The greatest difference between 35S:ABF3 and control plant lines in the percentage of genes belonging to a particular functional category was 1.3% for the 'other metabolic processes' category at the 2 h time point. This suggests that the overall functional response of 35S:ABF3 and control plant lines at the gene expression level was similar.

**Figure 3:**
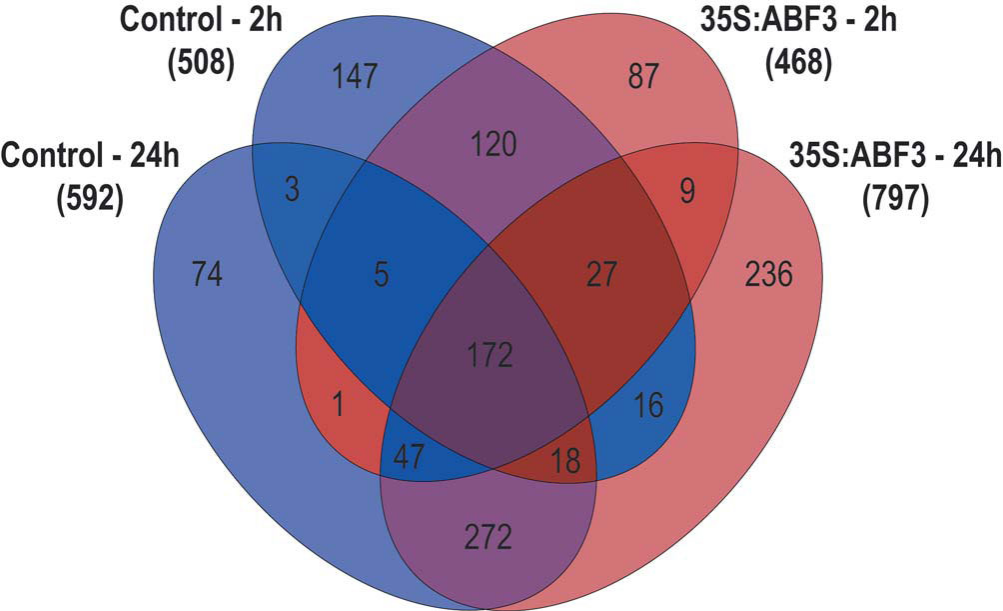
**Overlap of differentially expressed genes in 35S:ABF3 and control plants in response to drought**. Four-way Venn diagram showing the overlap of differentially expressed genes in 35S:ABF3 and control plant lines in response to drought at 2 h and 24 h. Regions corresponding to genes that are regulated commonly in both plant lines are shaded in purple. Regions corresponding to genes that show enhanced regulation in 35S:ABF3 plants are shaded in red and those that show attenuated regulation in 35S:ABF3 plants are shaded in blue.

In total, 1234 genes were differentially expressed in at least one plant line during at least one time point. The overlap in genes expressed in the two plant lines at the two different time points is depicted with a four-way Venn diagram in Figure 3. These genes can be subdivided into three categories. There are 564 genes that were differentially expressed in both control and 35S:ABF3 lines at the same time points suggesting that they were commonly regulated in both lines. There are 407 genes that are regulated differently in control and 35S:ABF3 plant lines that show an enhanced response in the 35S:ABF3 line. Finally, there are 263 genes that are regulated differently in control and 35S:ABF3 plant lines that show an attenuated response in the 35S:ABF3 line. In the latter two categories, these genes are either uniquely differentially expressed in one line or the other, or they are differentially expressed in one line at one time point but not in the other line at that time point. Since only two time points were examined, it is difficult to determine if the observed differences in the two lines are due to differences in the timing of gene expression or in the magnitude of gene expression or some combination of both of these factors. Additional file 3 contains a list of all of the differentially expressed genes found in each of the three categories.

**Figure 4:**
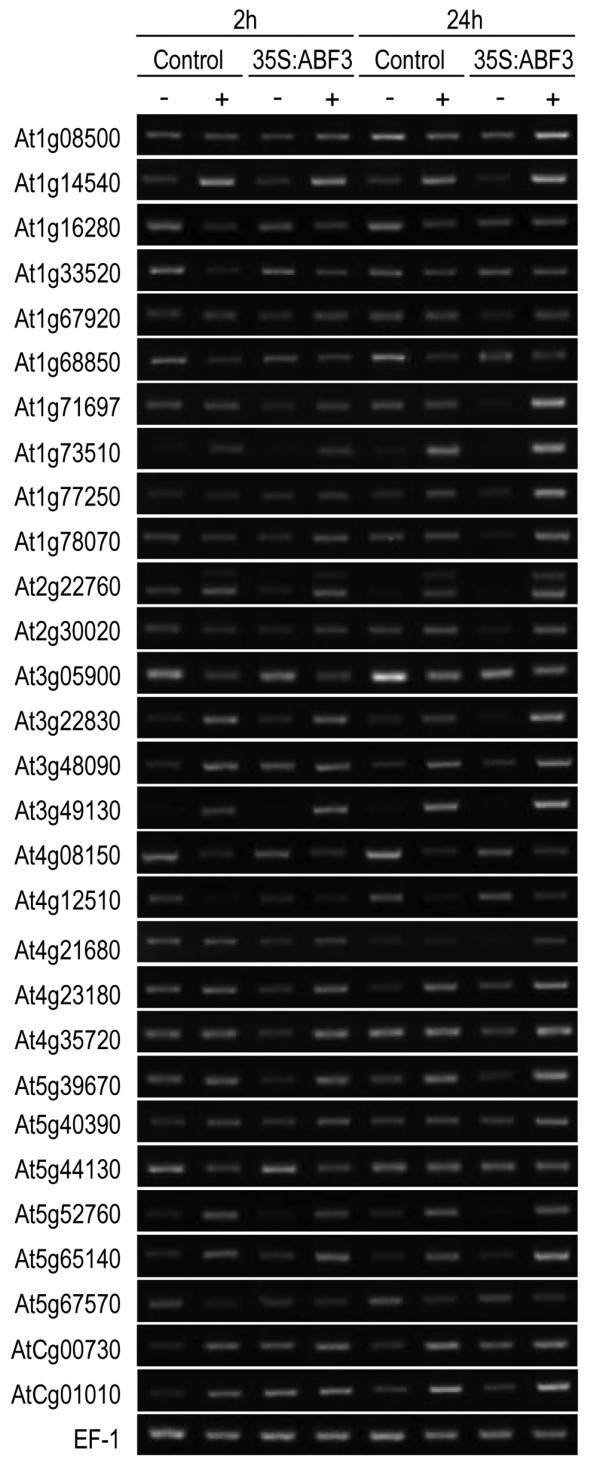
**RT-PCR confirmation of microarray data**. RT-PCR was performed using RNA from unstressed (-) or drought stressed (+) control and 35S:ABF3 plants at either the 2 h or 24 h time point. Genes showing either enhanced or attenuated regulation in 35S:ABF3 plants in response to drought were amplified using gene-specific primers. *Elongation factor 1-α *(*EF-1*) was amplified as an internal control.

In order to confirm the microarray results, RT-PCR was performed on 32 genes from the enhanced and attenuated categories. Twenty-nine of the examined genes exhibited expression patterns that were consistent with the microarray results, confirming the reliability of the microarray data (Figure 4). Amplification of one gene, At2g22760, produced two bands, one corresponding to the expected size of 526 bp. The primers for this gene were designed around an intron and the size of the second band is similar to the 664 bp that would be expected if the intron were not spliced, suggesting that this band may represent a splice variant.

### Genes commonly regulated in both 35S:ABF3 and control lines

There are 564 genes that are commonly regulated in both 35S:ABF3 and control lines. Of these, 172 show differential expression in both lines at both time points, 120 are only differentially expressed at 2 h and 272 are only differentially expressed at 24 h.

A number of genes in this category are known to act in pathways that are upstream or independent of ABF3, which is consistent with their common pattern of regulation in both 35S:ABF3 and control plant lines (Table 3). Since ABF3 is an ABA-dependent transcription factor, ABA biosynthesis should occur upstream of ABF3 activity. Consistent with this, *AtNCED3 *(At3g14440), which encodes a 9-*cis*-epoxycarotenoid dioxygenase enzyme involved in ABA biosynthesis, was upregulated in both lines at both time points in response to drought treatment as was the gene *CYP707A3 *(At5g45340), which encodes a cytochrome P450 monooxygenase involved in ABA catabolism.

**Table 3:** Genes commonly regulated in 35S:ABF3 and control plant lines that act in pathways upstream or independent from ABF3

			2 h		24 h	
Probe Set ID	AGI	Annotation	Control	35S:ABF3	Control	35S:ABF3
***ABA Metabolism***						
257280_at	At3g14440	AtNCED3	3.653	4.141	2.385	2.834
248964_at	At5g45340	CYP707A3	2.007	2.192	3.177	3.399
***DREB Transcription Factors***						
250781_at	At5g05410	DREB2A	3.945	4.672	4.755	4.602
256430_at	At3g11020	DREB2B	1.376	1.562	1.94	1.987
***NAC Transcription Factors***						
260203_at	At1g52890	ANAC019	3.945	4.409	4.264	4.608
258395_at	At3g15500	ANAC055/AtNAC3	3.088	3.258	2.297	2.557
253872_at	At4g27410	ANAC072/RD26	2.602	3.183	2.567	2.594

Values are log2 fold changes.

Several transcription factors have been identified that mediate drought response pathways independent from the ABF3 pathway. Members of the DREB family of transcription factors function in abiotic stress signalling and members of the DREB2 subfamily are known to function in ABA-independent drought stress signalling [59]. *DREB2A *(At5g05410) and *DREB2B *(At3g11020), as expected, were upregulated in response to drought in both plant lines at both time points (Table 3). In addition, *DREB1B/CBF1 *(At4g25490) and *DREB1C/CBF2 *(At4g25470) were also upregulated in both lines at both time points and DDF1 (At1g12610) was upregulated at 24 h (Additional file 3). While the DREB1 subfamily is primarily associated with cold stress signalling [59], there is some evidence that members also function in dehydration stress [8,38,60,61].

Three NAC transcription factors also function in a drought signalling pathway that is independent from ABF3 [42]. *ANAC19 *(At1g52890), *ANAC055/ATNAC3 *(At3g15500), and *ANAC072/RD26 *(At4g27410) are all expressed at both time points in both plant lines (Table 3). Another six members of the NAC family of transcription factors (At1g01720, At1g69490, At1g77450, At2g02450, At3g49530, and At5g63790) are also commonly regulated in both 35S:ABF3 and control plant lines (Additonal file 3).

Also in this category are many genes that are known to be regulated in response to drought stress. This includes genes involved in the biosynthesis of osmolytes, late embryogenesis abundant proteins (LEA), kinases, phosphatases, and transcription factors, as well as several different types of transporters (Table 4). This further suggests that some drought signalling pathways are unaffected by *ABF3 *overexpression

**Table 4:** Examples of typical drought-responsive genes regulated similarly in 35S:ABF3 and control plant lines

			2 h		24 h	
Probe Set ID	AGI	Annotation	Control	35S:ABF3	Control	35S:ABF3
***Osmolyte metabolism***						
251505_at	At3g59050	Polyamine oxidase 3			1.365	1.409
266072_at	At2g18700	Trehalose-phosphatase/synthase 11 (AtTPS11)	1.173	1.290		
254321_at	At4g22590	Trehalose-6-phosphate phosphatase, putative			2.262	2.652
250467_at	At5g10100	Trehalose-6-phosphate phosphatase, putative			1.716	1.560
***LEA class proteins***						
250648_at	At5g06760	LEA group 1 domain-containing protein	2.281	2.823	2.537	3.013
262128_at	At1g52690	LEA protein, putative	3.087	3.667		
267261_at	At2g23120	Expressed protein			1.651	1.688
266392_at	At2g41280	LEA protein M10			2.258	2.473
252988_at	At4g38410	Dehydrin, putative			2.692	2.516
***Kinases***						
252592_at	At3g45640	MAPK, putative (MPK3)	1.681	1.772	1.259	1.651
258682_at	At3g08720	Serine/threonine protein kinase (PK19)			1.160	1.383
250673_at	At5g07070	CBL-interacting protein kinase 2 (CIPK2)	1.408	1.261	1.551	1.827
254996_at	At4g10390	Protein kinase family protein			2.177	1.763
249361_at	At5g40540	Protein kinase, putative			2.768	3.066
248821_at	At5g47070	Protein kinase, putative			1.547	1.487
***Phosphatases***						
247957_at	At5g57050	Abscisic acid-insensitive 2 (ABI2)	1.617	2.228		
247723_at	At5g59220	Protein phosphatase 2C, putative	1.675	2.115		
251259_at	At3g62260	Protein phosphatase 2C, putative	2.533	2.710	2.902	2.440
253323_at	At4g33920	Protein phosphatase 2C family protein	1.385	1.615	1.767	1.779
***Transcription factors***						
250582_at	At5g07580	Ethylene-responsive element-binding family protein	-1.878	-1.668		
265452_at	At2g46510	Basic helix-loop-helix family protein			1.918	2.460
247509_at	At5g62020	Heat shock transcription factor B2A (AtHSFB2A)	1.133	1.135	1.594	1.900
251272_at	At3g61890	Homeobox-leucine zipper protein 12 (HB-12)	2.421	2.536	1.914	2.554
260237_at	At1g74430	Myb family transcription factor (MYB95)			1.931	1.942
255753_at	At1g18570	Myb family transcription factor (MYB51)	2.127	1.409		
254652_at	At4g18170	WRKY family transcription factor (WRKY28)	3.564	2.709		
261648_at	At1g27730	Zinc finger (C2H2 type) family protein (ZAT10)	3.665	3.663	4.978	5.127

Values are log2 fold changes.

### Genes with an enhanced response in 35S:ABF3 plants

There are 407 genes that demonstrated an enhanced response in 35S:ABF3 plants. Of these, 332 are uniquely regulated in 35S:ABF3 plants with 9 differentially expressed at both time points, 87 differentially expressed only at 2 h and 236 differentially expressed at 24 h. In addition, 47 genes are differentially expressed in the control line at only 24 h and 27 genes are differentially expressed in the control line at only 2 h while in the 35S:ABF3 line they are differentially expressed at both time points. Finally, there is a transposable element gene that is differentially expressed in 35S:ABF3 plants at 2 h but is only differentially expressed in control plants at 24 h.

A number of genes in this category could contribute to the enhanced drought tolerance of 35S:ABF3 plants (Table 5). Several genes involved in the biosynthesis of the osmolyte trehalose show enhanced upregulation while two genes involved in osmolyte catabolism show enhanced downregulation. Two genes involved in detoxification show enhanced upregulation in 35S:ABF3 as do three LEA proteins. A number of transporters also show enhanced regulation in 35S:ABF3 lines.

**Table 5:** Genes showing an enhanced response in 35S:ABF3 plants that may contribute to drought-tolerance

			2 h		24 h	
Probe Set ID	AGI	Annotation	Control	35S:ABF3	Control	35S:ABF3
***Osmolyte metabolism***						
248404_at	At5g51460	Trehalose-6-phosphate phosphatase (TPPA)				1.402
247228_at	At5g65140	Trehalose-6-phosphate phosphatase, putative	1.479	1.587		2.224
263452_at	At2g22190	Trehalose-6-phosphate phosphatase, putative		1.250		
254806_at	At4g12430	Trehalose-6-phosphate phosphatase, putative		1.146		
252983_at	At4g37980	Mannitol dehydrogenase, putative/elicitor activated gene 3 (ELI3-1)				-1.061
251729_at	At3g56310	Alpha-galactosidase, putative				-1.160
***Detoxification***						
266299_at	At2g29450	Glutathione S-transferase (class tau) 5 (AtGSTU5)				2.268
258665_at	At3g08710	Thioredoxin H-type 9 (ATH9)				1.188
***LEA class proteins***						
259516_at	At1g20450	Dehydrin/early response to dehydration 10 (ERD10)				1.584
252102_at	At3g50970	dehydrin/XERO2/low-temperature-induced protein				2.800
265211_at	At2g36640	LEAprotein/embryonic cell protein 63 (AtECP63)		1.652		
***Transporters***						
249063_at	At5g44110	ABC transporter family protein		1.017	1.540	1.826
263918_at	At2g36590	Proline transporter 3 (ProT3)		1.907		1.845
245868_at	At1g58030	Cationic amino acid transporter 2 (CAT2)				-1.406
260543_at	At2g43330	Inositol transporter 1 (AtINT1)		1.118		0.160
262756_at	At1g16370	Organic cation/carnitine transporter 6 (AtOCT6)/carbohydrate transmembrane transporte				1.992
245499_at	At4g16480	Inositol transporter 4 (AtINT4)				-1.031
254291_at	At4g23010	UDP-galactose transporter 2 (AtUTR2)				1.081
267423_at	At2g35060	K+ uptake permease 11 (KUP11)/potassium transporter family protein				-1.202
249298_at	At5g41330	Potassium channel tetramerisation domain-containing protein				-1.157
256402_at	At3g06130	Heavy-metal-associated domain-containing protein				-1.363
247128_at	At5g66110	Heavy-metal-associated domain-containing protein				-1.716
261143_at	At1g19770	Purine permease 14 (AtPUP14); purine transmembrane transporter	1.222	1.571		1.569
262649_at	At1g14040	EXS family protein/ERD1/XPR1/SYG1 family protein				1.103
250151_at	At5g14570	High affinity nitrate transporter 2.7 (AtNRT2.7)				-1.317
251916_at	At3g53960	Proton-dependent oligopeptide transport (POT) family protein		1.278		1.548
252589_s_at	At3g45650/At3g45660	Proton-dependent oligopeptide transport (POT) family protein		1.164		0.740
254396_at	At4g21680	Proton-dependent oligopeptide transport (POT) family protein				2.426

Values are log2 fold changes.

Direct targets of ABF3 are likely found in this category of genes. In addition to showing enhanced regulation in 35S:ABF3 plants, it is expected that these genes possess at least one ABRE in their promoter. In addition, these genes are likely to be significantly differentially expressed at the 2 h time point. Amongst those genes showing an enhanced response in 35S:ABF3 plants that are significantly differentially expressed at the 2 h time point, 24 contain at least one ABRE according to the in *silico *analysis performed by Gómez-Porras et al [62] and are therefore identified as putative ABF3 targets (Table 6). Included in this group are four transcription factors, one choline kinase, one trehalose biosynthetic enzyme, one gene involved in ubiquitin-mediated protein degradation, and two transporters as well as seven other genes with undefined roles in the drought response and eight unknown genes.

**Table 6:** Potential targets of ABF3

	AGI			2 h		24 h	
Probe Set ID		Annotation	ABREs^1^	Control	35S:ABF3	Control	35S:ABF3
***Osmolyte biosynthesis***							
254806_at	At4g12430	Trehalose-6-phosphate phosphatase, putative	1		1.146		
***Protein degradation***							
262164_at	At1g78070	WD-40 repeat family protein	1		2.072	2.312	2.647
***Signalling***							
261506_at	At1g71697	Choline kinase, putative (AtCK1)	1	1.519	1.181		2.909
***Transcription***							
253259_at	At4g34410	AP2 domain-containing transcription factor, putative	2		3.865	5.126	4.940
259432_at	At1g01520	Myb family transcription factor	3	2.367	2.762		3.247
262098_at	At1g56170	Nuclear factor Y, subunit C2 (NF-YC2)	3		1.944		
261892_at	At1g80840	WRKY family transcription factor (WRKY40)	2		3.762	4.724	5.138
***Transporters***							
260543_at	At2g43330	Inositol transporter 1 (AtINT1)	1		1.118		
251916_at	At3g53960	Proton-dependent oligopeptide transport (POT) family protein	2		1.278		1.548
***Other***							
251904_at	At3g54130	Josephin family protein	1		1.043		1.311
265634_at	At2g25530	AFG1-like ATPase family protein	2				1.399
252557_at	At3g45960	Expansin family protein (EXPL3)	1		1.467		1.850
253217_at	At4g34970	Actin-depolymerizing factor, putative	1		-1.417	-2.589	-2.791
262126_at	At1g59620	Disease resistance protein (CC-NBS class), putative	2		1.235		
247246_at	At5g64620	Invertase/pectin methylesterase inhibitor family protein	1		-1.125		
246495_at	At5g16200	50S ribosomal protein-related	1		1.193	2.080	2.162
***Unknown***							
261193_at	At1g32920	Expressed protein	1		1.541	1.793	2.457
260227_at	At1g74450	Expressed protein	3		1.755	1.840	2.170
253155_at	At4g35720	Expressed protein	3		2.671		2.010
261065_at	At1g07500	Expressed protein	2		1.201		
256069_at	At1g13740	Expressed protein	5		2.090		
260367_at	At1g69760	Expressed protein	1		-1.278		
260005_at	At1g67920	Expressed protein	1	2.251	2.051		2.825
254356_at	At4g22190	Expressed protein	2	-1.118	-1.031		-1.423

Values are log2 fold changes.

^1^Number of ABA-responsive elements (ABREs) in the promoter region as determined by Gómez-Porras et al. [62]

Amongst those genes that show an enhanced response in 35S:ABF3 plants, there is an enrichment of genes that function in RNA processing pathways (Table 7). There are 8 genes involved in RNA processing that are similarly regulated in both control and 35S:ABF3 plants lines, 8 genes that show an attenuated response in 35S:ABF3, while 18 genes show an enhanced response. Many of these appear to be uniquely downregulated in 35S:ABF3 plants at 24 h. Eleven of these are predicted to function in RNA splicing or to be associated with the RNA splicing machinery while the others have roles in nucleocytoplasmic transport, deadenylation, or the function is not specifically known. Several genes that function in RNA processing have been found to function in ABA and abiotic stress signalling pathways [63-66]. This suggests that the differential regulation of RNA processing genes in 35S:ABF3 lines could contribute to its enhanced drought tolerance.

**Table 7:** Genes showing enhanced regulation in 35S:ABF3 with predicted function in RNA processing

			2 h		24 h	
Probe Set ID	AGI	Annotation	Control	35S:ABF3	Control	35S:ABF3
***Splicing and splicing-related***						
262110_at	At1g02840	Pre-mRNA splicing factor SF2/SR1 protein				-1.731
263035_at	At1g23860	Splicing factor RSZp21 (RSZP21)				-1.272
262295_at	At1g27650	U2 snRNP auxiliary factor small subunit, putative (AtU2AF35A)				-1.129
262931_at	At1g65700	Small nuclear ribonucleoprotein, putative				1.386
267102_at	At2g41500	LACHESIS (LIS)/related to yeast splicing factor PRP4				-1.260
252182_at	At3g50670	U1 small nuclear ribonucleoprotein 70 (U1-70k)				-1.525
251798_at	At3g55460	SC35-like splicing factor, 30 kD (SCL30)				-1.048
253668_at	At4g30220	Small nuclear ribonucleoprotein F (RUXF), putative				1.057
249870_at	At5g23080	SWAP domain-containing protein/TOUGH (TGH)				-1.476
246924_at	At5g25060	RNA recognition motif (RRM)-containing protein				-1.043
248369_at	At5g52040	Arginine/serine-rich splicing factor RSP41 (RSP41)				-1.009
***Nucleocytoplasmic transport***						
257817_at	At3g25150	Nuclear transport factor 2 (NTF2) family protein				-1.316
***Deadenylation***						
252679_at	At3g44260	CCR4-NOT transcription complex protein, putative		2.108	3.479	3.354
***Other***						
262804_at	At1g20880	RNA recognition motif (RRM)-containing protein				-1.335
261988_at	At1g33680	KH domain-containing protein				-1.123
254355_at	At4g22380	Ribosomal protein L7Ae/L30e/S12e/Gadd45 family protein				1.617
246088_at	At5g20600	Expressed protein				-1.145
247004_at	At5g67570	Pentatricopeptide (PPR) repeat-containing protein				-1.349

Values are log2 fold changes.

Interestingly, a member of the DREB family of transcription factors is also included in this group. *DREB1D/CBF4 *(At5g51990) was upregulated in 35S:ABF3 plants at both time points but was only upregulated in control plants at 24 h. DREB1D/CBF4 functions in both cold and drought stress signalling and, unlike the DREB2 subfamily, its expression is induced by ABA [38]. This suggests that CBF4 may act downstream of ABF3 in the ABA-dependent drought signalling pathway.

### Genes with an attenuated response in 35S:ABF3 plants

There are 263 genes that show an attenuated response in 35S:ABF3 plants. Of these, 224 are uniquely regulated in control lines with 3 differentially expressed at both time points, 147 differentially expressed only at 2 h and 74 differentially expressed only at 24 h. In addition, 18 genes are differentially expressed in the 35S:ABF3 line at only 24 h and 5 genes are differentially expressed in the 35S:ABF3 line at only 2 h while in the control line they are differentially expressed at both time points. Finally, there are 16 genes that are differentially expressed in the control line at 2 h and in the 35S:ABF3 line at 24 h.

Surprisingly, a number of genes that typically function in conferring drought tolerance show an attenuated response in 35S:ABF3 plants. This includes several peroxidases and glutaredoxins involved in detoxification, two genes involved in biosynthesis of the osmolytes raffinose and spermine, a heat shock protein, and a number of transporters (Additional file 3). Similarly a number of transcription factors and other signalling components also show an attenuated response in 35S:ABF3 plants. The reason for the attenuated response of these genes in 35S:ABF3 plants is not clear but might reflect differences in the physiological state of 35S:ABF3 plants due to their enhanced drought resistance.

A number of genes encoded by the chloroplast and mitochondrial genomes show an attenuated response in 35S:ABF3 plants (Table 8). Some of these genes encode proteins with electron transport activity or NADH dehydrogenase activity while others are predicted to function in transcription and translation processes. Most of these genes are upregulated in control lines only at the 2 h time point. The reason for the exclusive upregulation of these genes in the control line is not clear. The chloroplast NADH dehydrogenase (NDH) complex is predicted to be involved in cyclic electron transport around photosystem I, thereby dissipating energy and maintaining ATP supply under conditions of low CO2 availability following stomatal closure in response to stress [67]. The NDH complex may also be involved in detoxification in the chloroplast [68]. The upregulation of genes encoding NADH dehydrogenases may therefore reflect the greater sensitivity of the control plant line to stress. Rice plants overexpressing *ABF3 *were able to maintain higher photochemical efficiency during drought stress [11]. This suggests that one aspect of the drought tolerance conferred by *ABF3 *overexpression may be a minimization of the negative impact of drought stress on photosynthesis, which is reflected by differences in gene expression in the chloroplast. Similar effects may also occur in the mitochondria.

**Table 8:** Genes encoded by the chloroplast and mitochondrial genomes showing attenuated regulation in 35S:ABF3 plants

Probe Set			2 h		24 h	
ID	AGI	Annotation	Control	35S:ABF3	Control	35S:ABF3
***Electron transport activity***						
266045_s_at	At2g07727/AtMg00220	Cytochrome b (MTCYB) (COB) (CYTB)/apocytochrome b (cob) ^†^	2.060			
244903_at	AtMg00660	Hypothetical protein (orf149)^†^	1.645			
244977_at	AtCg00730	Subunit IV of cytochrome b6/f complex (petD)*	2.993		3.118	
***NADH dehydrogenases***						
244943_at	AtMg00070	NADH dehydrogenase subunit 9 (nad9)^†^	1.338			1.416
257337_at	AtMg00060	NADH dehydrogenase subunit 5 (nad5) (Transplice part 3 of 3)^†^	1.413			
244933_at	AtCg01070	NADH dehydrogenase ND4L (ndhE)*	1.733			
244994_at	AtCg01010	Chloroplast encoded NADH dehydrogenase unit (ndhF)*	3.240			
244934_at	AtCg01080	NADH dehydrogenase ND6 (ndhG)*	2.465			
244991_s_at	AtCg00890/AtCg01250	NADH dehydrogenase ND2 (ndhB)*	3.528			
***RNA processing***						
244999_at	AtCg00190	RNA polymerase subunit beta (rpoB)*	2.792			
***Translation***						
245005_at	AtCg00330	Chloroplast ribosomal protein s14 (rps14)*	3.796		3.644	
244970_at	AtCg00660	Ribosomal protein L20 (rpl20)*	2.379			
244939_at	AtCg00065	Ribosomal protein s12 (Transplice part 1 of 2) (rps12.1)*	1.653			
***ATPase subunits***						
244995_at	AtCg00150	Subunit of ATPase complex CF0 (atpI)*	2.049			
266012_s_at	AtMg00410/AtMg01170/At2g07699/At2g07741	ATPase subunit 6^†^	1.924			
***Other***						
257319_at	AtMg01100	Hypothetical protein (orf105a)^†^	1.181			
244989_s_at	AtCg00860/AtCg01280	Expressed protein (ycf2)*	3.493			
245008_at	AtCg00360	Protein required for photosystem I assembly and stability (ycf3)*	2.371			
244990_s_at	AtCg00870/AtCg01270	Hypothetical protein (ycf15/orf77)*	3.607			2.841
245016_at	AtCg00500	Acetyl-CoA carboxylase carboxyl transferase subunit beta (accD)*	1.452			
266014_s_at	At2g07722/AtMg00170/AtMg00620	Hypothetical protein^†^	1.987			

Values are log2 fold changes.

* indicates gene is encoded by the chloroplast genome.

^† ^indicates gene is encoded by the mitochondrial genome.

Four transposable element genes were also included in the attenuated response category (Table 9). Only one other transposable element gene was significantly differentially expressed and it was included in the enhanced regulation category. Many transposable elements are transcriptionally activated in response to stress conditions [69,70]. The attenuated regulation of transposable element genes in 35S:ABF3 plants might suggest that they are experiencing a lower level of stress that is insufficient to activate the transposable element genes, consistent with the drought tolerance of these plants.

**Table 9:** Transposable element genes showing attenuated regulation in 35S:ABF3 plants

Probe Set			2 h		24 h	
ID	AGI	Annotation	Control	35S:ABF3	Control	35S:ABF3
***Transposable element genes***						
265709_at	At2g03540	Transposable element gene			1.305	
263769_at	At2g06390	Transposable element gene	1.511			
257777_x_at	At3g29210	Transposable element gene	1.117			
257345_s_at	At3g33066	Transposable element gene; gypsy-like retrotransposon family (Athila)	2.379			

Values are log2 fold changes.

Interestingly, *DREB1A/CBF3 *showed an attenuated response in 35S:ABF3 plants. *DREB1A/CBF3 *was upregulated in control plant lines at both time points but was only upregulated in 35S:ABF3 plants at 2 h. This may again reflect differences in the tolerance of 35S:ABF3 and control plants to the drought stress, with control plants requiring a stronger or longer activation of *DREB1A/CBF3 *expression in response to the increased stress.

## Discussion

### The Impact of Cre recombinase on the transcriptome is minimal

In order to eliminate position effects and focus on unintended pleiotropic effects of transcription factor overexpression, the Cre/*lox *recombination system was employed to create a series of control plant lines that contain the selectable marker at the site of transgene insertion but from which the *ABF3 *transgene was excised. The use of the Cre/*lox *recombination system also allowed us to determine the impact of Cre recombinase on the transcriptome.

In tomato, petunia, tobacco, and to a lesser extent *Arabidopsis*, expression of Cre recombinase has resulted in abnormal phenotypes, including leaf chlorosis, stunted growth, and sterility [49,50]. Similarly, expression of Cre recombinase resulted in reduced proliferation and chromosomal abnormalities in cultured embryonic mouse cells [55,57], toxicity in dividing cells of *Drosophila melanogaster *[54], and chromosomal rearrangements in mouse spermatids leading to male sterility [56]. These abnormal effects of Cre recombinase are suspected to result from Cre-mediated recombination using cryptic *lox *sites that may be found in eukaryotic genomes. Cryptic *lox *sites that can be recognized by Cre recombinase have been identified in the genomes of yeast and humans as well as the chloroplast genome of tobacco [52,53,71,72].

If cryptic *lox *sites exist in plant genomes, expression of Cre recombinase could induce deletions or inversions of genome segments or even chromosome translocations that could adversely impact the plant. These deletions or inversions may alter the expression of genes found within this segment, which would be detectable by microarray. In three independent plants lines in which Cre recombinase was employed to excise the *ABF3 *transgene from the T-DNA insertion, only a small number of genes were found to be differentially expressed (Table 1). Of these, two genes were found to be differentially expressed in two out of the three control lines. The two genes are the chloroplast encoded *rps7 *(AtCg00900 and AtCg01240) and *rps12.1 *(AtCg00065) genes. In tobacco, one of the cryptic *lox *sites identified in the chloroplast genome is found just downstream of the start site of the *rps12.2/rps7 *operon [52,53]. The altered expression of *rps7 *might suggest that this cryptic *lox *site is conserved in *Arabidopsis *and may have undergone Cre-mediated recombination. Cre recombinase may therefore also be able to act on cryptic *lox *sites in the *Arabidopsis *chloroplast genome, resulting in a change in expression of the affected genes. It is, however, unclear how Cre recombinase is targeted to the chloroplast since it is only predicted to be targeted to the nucleus. Cre-mediated chloroplast genome deletions are not likely to be of great concern since chloroplast genomes containing deletions in essential genes are typically rapidly lost due to selection pressures [73-75], especially once the Cre recombinase has been removed. The impact of Cre recombinase on the nuclear transcriptome was negligible, which demonstrates that in *Arabidopsis *this technology does not produce unintended effects.

### The activity of ABF3 is strictly controlled

Microarray analysis of *Arabidopsis *plants overexpressing the transcription factor *ABF3 *suggests that alterations to the transcriptome are minimal when position effects are eliminated as a source of variation. In the absence of stress, a small number of genes were differentially expressed in three 35S:ABF3 plant lines (Table 2), but no genes were differentially expressed in more than one independent line.

Members of the ABF/AREB family of transcription factors bind to ABREs found in the promoters of ABA-responsive genes [29,31]. If the genes identified by microarray analysis are actual downstream targets of ABF3, they would be expected to contain at least one ABRE. An *in silico *analysis of the *Arabidopsis *nuclear genome has identified 3829 genes containing one or more ABREs [62]. None of the nuclear genes identified by microarray analysis of 35S:ABF3 transgenic plants are predicted to contain an ABRE. Other members of the ABF/AREB subfamily of transcription factors localize to the nucleus [76,77], and it is likely that ABF3 similarly functions in the nucleus. Therefore, it is unlikely that the chloroplast genes identified by microarray analysis are functional targets of ABF3. This suggests that overexpression of *ABF3 *alone is not sufficient to alter the transcriptome.

This result was unexpected as previous work identified a number of genes with altered expression in *Arabidopsis *[7] and rice [11] overexpressing *ABF3*. Similarly, overexpression studies of many other transcription factors have revealed alterations in gene expression and this approach is typically used to identify the gene network controlled by that particular transcription factor [39,42,58]. The absence of differentially expressed genes in 35S:ABF3 transgenic plants suggests that an additional signal is required to activate ABF3 that is not present in unstressed plants.

There is accumulating evidence that members of the ABF/AREB family of transcription factors are regulated by phosphorylation. ABF2/AREB1 transactivation of a reporter gene in the presence of ABA was inhibited by the addition of the protein kinase inhibitor staurosporine [31] and ABI5 is phosphorylated following ABA treatment [78]. Several studies have suggested a role for members of the SnRK2 family of protein kinases in the phosphorylation of ABF/AREB transcription factors [79-84]. ABF3 and ABF4/AREB2 interact with the calcium-dependent protein kinase AtCPK32 and evidence suggests that it phosphorylates a highly conserved serine residue in ABF4/AREB2 that is necessary for activity [85]. The protein kinases CPK4 and CPK11 are also likely to phosphorylate ABF1 and ABF4/AREB2 and their activity is enhanced by ABA [86]. It is possible that in the absence of stress, ABF3 is not phosphorylated and therefore cannot activate gene expression.

Furthermore, other factors necessary for the activity of ABF3 may not be expressed in the absence of abiotic stress. Members of the ABF/AREB family have been shown to interact with diverse proteins that are predicted to modulate their transcriptional activity. ABF2/AREB1 interacts with an arm-repeat protein that is predicted to positively regulate its activity [87]. ABI5, ABF1, ABF3, and ABF4/AREB2 can interact with the transcription factor ABI3 [88,89]. Furthermore, the *rd29a *promoter contains both an ABRE as well as a dehydration-responsive elements that is bound by members of the DREB/CBF family of transcription factors and the two elements function interdependently to activate expression of *rd29a *[90]. Members of the ABF/AREB can also heterodimerize [91], suggesting that other members of this family may need to be expressed in order for ABF3 to be functional. Therefore, it is possible that other components of the stress response pathway are necessary in order for ABF3 to be active, preventing ABF3 from altering gene expression in the absence of stress.

While the 35S:ABF3 plants did not show any changes in transcription in the absence of drought stress, there were some phenotypic differences compared to control plants. Most notably, the 35S:ABF3 plants were smaller in size than control plants of the same age, with the difference becoming more pronounced with increased age (Figure 2B, C). This is a common observation for plants overexpressing transcription factors [7,33,39,60] and is often overcome by using tissue-specific or inducible promoters [32,60]. At least some of the growth retardation may be attributable to reduced transpiration rates of 35S:ABF3 plants compared to control plants (Figure 2A), which is consistent with the observation that *Arabidopsis *plants overexpressing *ABF3 *typically have stomata with smaller openings than do wild-type plants [7]. This would suggest that *ABF3 *may govern gene networks involved in stomatal closure. Consistent with this, ABF3 is expressed in guard cells and its expression is further induced in these cells in response to ABA [7,92]. Since our analysis was performed on whole plants, it is likely that changes in the transcriptional network of guard cells would not be readily detectable.

### Overexpression of *ABF3 *results in transcriptional reprogramming of the drought response

Overexpression of *ABF3 *confers drought tolerance to *Arabidopsis *plants and since ABF3 is a transcription factor, it can be predicted that this will occur through changes to the transcriptional network of the plants. Consistent with this, the expression profile of *Arabidopsis *plants overexpressing *ABF3 *differed from that of control plants. As might be expected, there were a number of genes with expression patterns that appeared to be enhanced in 35S:ABF3 plants compared to control plants. Whether this occurred through alterations in the timing or strength of expression could not be established with the two time points considered in this study. Many of the genes with enhanced expression are known to function in mitigating drought stress, suggesting that they could contribute incrementally to the enhanced drought tolerance of 35S:ABF3 plants (Table 5). Those genes showing enhanced regulation in 35S:ABF3 plants likely include some direct targets of ABF3. In particular, those genes that are differentially expressed at 2 h and contain at least one ABRE are the most likely targets of ABF3 (Table 6).

Interestingly, there seemed to be a number of genes involved in RNA processing that showed enhanced expression in 35S:ABF3 plant lines. Most of these were downregulated at the 24 h time point. A number of RNA processing mutants impaired in ABA response have demonstrated the importance of RNA processing to ABA signalling [93]. Loss-of-function mutations in two genes encoding subunits of a nuclear cap-binding complex cause ABA hypersensitivity [63,66] as do mutations in a pre-mRNA splicing factor that is important for both mRNA splicing and turnover [94], a poly(A)-specific ribonuclease that is predicted to function in mRNA degradation [65], and a protein with homology to the Sm-like small nuclear ribonucleoproteins (snRNPs) that function in mRNA splicing, export, and degradation [95]. Two phosphatases that belong to the family of proteins that dephosphorylate the C-terminal domain of RNA polymerase II negatively regulate stress-responsive gene transcription [96] and a mutation in one of these results in decreased ABA sensitivity [97]. The enrichment of genes involved in RNA processing that show enhanced expression in the 35S:ABF3 line might suggest that ABF3 plays an important role in the regulation of ABA-responsive RNA processing events. The downregulation of many of the RNA processing genes is consistent with the negative regulatory role observed for several of the RNA processing proteins previously identified in the ABA-signalling pathways.

In addition to those genes showing enhanced regulation in 35S:ABF3 plants, there were also a number of genes with attenuated expression. Many of these are known to encode proteins with roles in minimizing drought stress and their attenuated expression is not consistent with the drought tolerance of the 35S:ABF3 plants. These genes may be reflective of a greater transcriptional reprogramming in 35S:ABF3 plants than merely enhancing the rate or level of expression of a subset of genes. The strong activation of the ABF3 pathway may result in co-ordinated feedback that modulates other drought responsive pathways, resulting in attenuated gene expression in some cases. This might reflect cooperativity between some of the drought signalling pathways. In many cases, drought-responsive transcription factors have been shown to function in concert to activate gene expression [39,40,43,90]. Furthermore, it has been observed that downregulation of the phosphoinositide pathway in *Arabidopsis *results in an upregulation of the *DREB2A *gene as well as several DREB2A-regulated genes, suggesting a negative interaction between two drought signalling pathways [98].

The degree of stress experienced by the control plants should be greater than that experienced by the 35S:ABF3 plants and this may ultimately have secondary consequences on the transcriptional network that is reflected by the genes showing an attenuated transcriptional response. This is consistent with the observation that several transposable element genes show attenuated expression in 35S:ABF3 plants (Table 9). Many transposons are activated in response to stress [69,70] and their delayed activation in 35S:ABF3 plants could be a result of the drought tolerance of these plants.

Similarly, the enrichment of genes encoded by the chloroplast and mitochondrial genomes in the attenuated category (Table 8) may also reflect the greater drought sensitivity of the control plant lines. Drought has a significant and complex impact on the activities of both the mitochondria and chloroplasts and this is reflected in changes in gene expression [99,100]. Several of the chloroplast and mitochondria-encoded genes showing attenuated expression encode NADH dehydrogenases. The chloroplast NDH complex is predicted to function in cyclic electron transport around photosystem I to help dissipate excess energy during abiotic stresses such as drought and thereby to alleviate oxidative stress [67]. The elevated expression of chloroplast NDH complex subunits and other chloroplast and mitochondria-encoded genes in control plants may therefore be indicative of the increased stress of these plants.

Drought stress can change the composition of a plant, altering levels of oils, proteins and other constituents, which can affect the commercial and nutritional value of the plant [101-107]. In some cases, drought can also initiate the accumulation of higher levels of dangerous toxins and anti-nutrients [102,105,108]. The altered expression of transposable element genes and genes that are encoded by the chloroplast and mitochondrial genomes in control plants suggests that these plants are experiencing a higher level of drought stress than 35S:ABF3 plants. It is therefore possible that control plants exposed to drought will exhibit more compositional changes compared to unstressed plants than 35S:ABF3 plants. This is an important consideration because compositional analysis is one of the parameters used to determine the substantial equivalence of transgenic plants to their non-transgenic comparators during the risk assessment process. These results might suggest that 35S:ABF3 plants are better able to maintain compositional standards under drought stress than their non-transgenic counterparts.

### Overexpression of *ABF3 *does not activate unintended gene networks during the drought response

While differences were observed in the patterns of gene expression of 35S:ABF3 and control plant lines, at the functional level the response was very similar. Functional categorization of the significantly differentially expressed genes demonstrated that the percentage of genes in each category was relatively similar between the two plant lines (Additional file 2). This suggests that overexpression of *ABF3 *does not activate new gene networks but simply functions to modify existing gene networks that function in drought response. Although there were genes that were uniquely regulated in each of the two plant lines, many of these genes did observe a change in expression in the other plant line that was not of a high enough magnitude to meet our criteria for differential expression (Additional file 3). This suggests that these genes are showing stronger and/or earlier differential regulation as opposed to being uniquely regulated.

Among the genes that were differentially regulated between the 35S:ABF3 and control plant lines, there was no indication that overexpression of *ABF3 *activated any unintended gene networks. Several closely related members of the ABF/AREB transcription factor family function in seed germination and seed and early seedling developmental pathways, including ABI5 whose role in these pathways has been best characterized [109-111]. Although ABF3 is primarily associated with abiotic stress signalling in vegetative tissues, there is some evidence that it may also function in seed and early seedling developmental processes, although its role may be relatively minor. Microarray data from GENEVESTIGATOR [112] indicates that ABF3 is expressed during seed development, although at low levels compared to ABI5. Also, levels of *ABF3 *are enhanced by dehydration and salinity stresses or by ABA treatment in germinating embryos [113] and an alternative splice form of the *ABF3 *gene was identified from a cDNA library prepared from immature seed [91]. Double mutant analysis has also revealed several redundancies between ABF3 and ABI5, including sensitivity to ABA during germination, stress sensitivity of root growth, resistance to glucose, and regulation of the ABA-induced vegetative expression of *RAB18 *and *RD29B *[88]. *ABI5 *and *ABF3 *also appear to antagonistically cross-regulate each other [88,114].

If ABF3 does function in seed developmental pathways, it is possible that altering its pattern of expression could ectopically activate gene networks involved in those pathways. Another member of the ABF/AREB family of transcription factors, ABF2/AREB1, which functions in abiotic stress signalling and glucose response, is also expressed in embryonic axes in dry siliques [33,77]. Overexpression of a phosphorylated active form of ABF2/AREB1 led to the activation of several seed storage protein genes in vegetative tissues, many of which also have binding sites for ABI3, another transcription factor involved in seed development [81]. However, none of the seed storage genes activated by the phosphorylated active form of *ABF2/AREB1 *were differentially expressed in the 35S:ABF3 plants, nor were *AtEm1 *and *AtEm6*, two LEA-class genes that are ABI5 targets expressed during seed maturation (data not shown). This further demonstrates that overexpression of *ABF3*, while modifying patterns of gene expression, did not activate unintended pathways in response to drought stress in *Arabidopsis*. This suggests that overexpression of a transcription factor to confer an abiotic stress tolerance trait may not necessarily produce unintended pleiotropic effects.

## Conclusions

The Cre/*lox *recombination system allowed us to create paired plant lines with identical T-DNA insertion sites either with or without the *ABF3 *transgene in order to eliminate position effects in our analysis of unintended effects. This approach also allowed us to examine unintended effects resulting from the expression of Cre recombinase. We found that Cre recombinase had a minimal impact on the transcriptome, which suggests that it produces few unintended effects in *Arabidopsis*. Microarray analysis of *Arabidopsis *plants overexpressing *ABF3 *demonstrated that the impact on the transcriptome is minimal. In the absence of drought stress, there were no differentially expressed genes. In response to drought stress, a reprogramming of the drought response was observed, suggestive of changes in the timing or strength of expression of some genes in 35S:ABF3 plants. Some of these changes may be directly related to the action of ABF3 while others may reflect an altered physiological state as a result of the enhanced drought tolerance of 35S:ABF3 plants. Amongst the differentially expressed genes, no unintended pathways appeared to be activated as a result of *ABF3 *overexpression. These results are significant because they demonstrate that plant responses to abiotic stresses such as drought may be strictly coordinated at multiple regulatory steps and this limits the extent of unintended pleiotropic effects. This demonstrates that engineering stress tolerance through manipulation of endogenous plant pathways may not necessarily produce unintended pleiotropic effects despite the complexity of such traits. This is an important finding for establishing the safety of such traits as they begin to enter the market in the near future.

## Methods

### Generation of transgenic plants

To generate 35S:ABF3 plant lines, *Arabidopsis thaliana *Col-0 plants were transformed by the floral dip method [115] using *Agrobacterium tumefaciens *strain GV3101 harbouring the pCAMBIA3300 vector containing the Cauliflower mosaic virus 35S promoter, *ABF3 *coding region, and *nopaline synthase *transcriptional terminator, bordered on either side *loxP *sites. Homozygous single insert transformants were identified by Southern blot and segregation analysis and three lines with high levels of *ABF3 *expression, as determined by RT-PCR analysis, were selected for further analysis.

To generate plant lines expressing *Cre *recombinase, *Arabidopsis thaliana *Col-0 plants were transformed as above with a pCAMBIA1200 vector containing the Cauliflower Mosaic Virus 35S promoter, *Cre *recombinase coding region, and *nopaline synthase *transcriptional terminator.

To generate control plant lines, 35S:ABF3 plants were crossed with plants expressing *Cre *recombinase in order to excise the *35S-ABF3-nos *transgene. The F1 plants were PCR genotyped to identify those that underwent successful excision, using the primers ABF-F (5'-ATGGGGTCTAGATTAAAC-3') and Nos-R (5'-CCGATCTAGTAACATAGATG-3'), which are specific for the *ABF3 *gene and the *nopaline synthase *transcriptional terminator, respectively. One plant from each cross, Control-48; Cre 1.1.2, Control-57; Cre 1.2.2 and Control-59; Cre 2.2.1, was selected from which loss of the *35S-ABF3-nos *transgene was confirmed by PCR (Additional File 1). F2 progeny from the selected F1 plants were then backcrossed to wild-type plants to eliminate *Cre *recombinase. The F1 plants from this cross were again PCR genotyped to identify those from which the Cre gene was lost, using primers CRE.F (5'-CCAGGCGTTTTCTGAGCATACCTG-3') and CRE.R (5'-CTCTGACCAGAGTCATCCTTAGCG-3'), which are both specific for the *Cre *recombinase gene. One plant from each cross, Control-48 1.1.2.5, Control-57 1.2.2.5 and Control-59 2.2.1.4, was selected from which loss of the *Cre *recombinase gene was confirmed by PCR (Additional File 1). The selected F1 plants represent the control plant lines and the F2 progeny of these plants were used in subsequent experiments.

### Growth of *Arabidopsis *plants

Seeds were surface sterilized by soaking for 20 min in 25% (v/v) commercial Clorox (final concentration of 1.3% sodium hypochlorite) and 0.05% (v/v) Triton X-100 (Fisher Scientific, Hampton, NH, USA), and then rinsing four times with distilled water. Seeds were germinated on MS media and grown at 22°C with a 16 h photoperiod. To determine the growth rate of plants, three-day-old seedlings were transplanted onto fresh MS plates and after one week or four weeks of growth the fresh weight of the seedlings was measured. To measure transpiration rate, leaves of a similar developmental stage were excised from four-week-old plants and the loss of weight over a 24 h-period was measured. For microarray analysis, seeds were germinated on MS media and one-week-old seedlings were collected. For the drought stress, seedlings were transferred onto paper towels and harvested after 2 and 24 h.

### Microarray Analysis

For each sample, three biological replicates were prepared. RNA was extracted from seedlings using the RNeasy Plant Mini Kit (Qiagen, Germantown, MD, USA), following the manufacturer's protocol. A 2100 Bioanalyser (Agilent Technologies, Santa Clara, CA, USA) was used to determine the quality of the RNA. Microarray analysis was performed using the GeneChip^® ^Arabidopsis ATH1 Genome Array (Affymetrix Inc., Santa Clara, CA, USA). Standard RNA processing, hybridization, and scanning protocols were followed as recommended by the GeneChip^® ^Expression Analysis Technical Manual (Affymetrix Inc., Santa Clara, CA, USA). Hybridization, and scanning were performed at Agriculture and Agri-Food Canada (Winnipeg, MB, Canada) by Mark Jordan. RMA procedure [116] was performed to normalize data using the AffylmGUI R software package from Bioconductor (http://www.bioconductor.org/)[117]. Analysis of differential expression was done using a moderated t-test with empirical Bayes smoothing [118]. Microarray data from this study have been deposited at ArrayExpress (accession number E-MEXP-2435).

### Reverse transcription-PCR (RT-PCR)

DNaseI-treated RNA was used for first strand cDNA synthesis using Superscript III reverse transcriptase (Invitrogen, Carlsbad, CA, USA) and oligo (dT)_18 _primers according to the manufacturer's protocol. Between 24 and 30 cycles of PCR amplification was performed using gene-specific primers. As an internal control, *elongation factor 1-α *(*EF-1*) was amplified. Sequences of all primers used in RT-PCR analysis can be found in Additional file 4.

## List of Abbreviations

ABF: ABA-responsive element binding factor; AREB: ABA-responsive element binding protein; ABA: abscisic acid; ABRE: ABA-responsive element; DREB: dehydration responsive element binding protein; CBF: C-repeat binding factor; NAC: NAM, ATAF1,2, CUC2; ZFHD: zinc finger homeodomain; CaMV 35S promoter: Cauliflower Mosaic Virus 35S promoter; RT-PCR: reverse transcription polymerase chain reaction; LEA proteins: late embryogenesis abundant proteins; NDH complex: NADH dehydrogenase complex; ABI: ABA-insensitive.

## Authors' contributions

AA participated in the design of the study and carried out the vector construction, plant transformation and molecular analysis, microarray analysis, and contributed to the drafting of the manuscript. JS participated in the final microarray analysis, carried out the RT-PCR work, and drafted the manuscript. BM conceived of the study and participated in its design and coordination, and participated in the drafting of the manuscript. All authors read and approved the final manuscript.

## Supplementary Material

Additional file 1 **PCR genotyping of control plant lines**. (A) Control plant lines, derived from 35S:ABF3 plants crossed with plants expressing *Cre *recombinase, were PCR genotyped to identify plants from which the *35S-ABF3-nos *transgene was excised. (B) Control plant lines following a backcross with Col-0 plants were genotyped to identify plants that lost the *Cre *gene by genetic segregation. Plant lines with the desired genotype that were selected following each cross are indicated with asterisks. Click here for file

Additional file 2 **Functional categorization of drought responsive genes in 35S:ABF3 and control plants**. Distribution of genes differentially expressed in 35S:ABF3 and control plant lines at 2 h and 24 h following drought stress into functional categories. Click here for file

Additional file 3 **Genes differentially expressed in response to drought in 35S:ABF3 and control plants**. This table includes a complete list of genes differentially expressed in both 35S:ABF3 and control plant lines in response to drought at 2 h and 24 h. Genes are divided into three groups including those that are similarly regulated in both 35S:ABF3 and control plants, those that show enhanced regulation in 35S:ABF3 plants, and those that show attenuated regulation in 35S:ABF3 plants. Click here for file

Additional file 4 **Sequences of primers used in RT-PCR analysis**. This table includes sequences of primers used to amplify selected genes in the RT-PCR analysis performed to confirm microarray results. Click here for file

## References

[B1] Boyer JS Plant productivity and environment Science 1982 218 443 448 10.1126/science.218.4571.443 17808529

[B2] Burke EJ Brown SJ Christidis N Modeling the recent evolution of global drought and projections for the twenty-first century with the Hadley Centre climate model J Hydrometeorology 2006 7 1113 1125 10.1175/JHM544.1

[B3] Meehl GA Washington WM Santer BD Collins WD Arblaster JM Hu A Lawrence DM Teng H Buja LE Strand WG Climate change projections for the twenty-first century and climate change commitment in the CCSM3 J Climate 2006 19 2597 2616 10.1175/JCLI3746.1

[B4] Century K Reuber TL Ratcliffe OJ Regulating the regulators: the future prospects for transcription-factor-based agricultural biotechnology products Plant Physiol 2008 147 20 29 10.1104/pp.108.117887 18443103 PMC2330319

[B5] Salmeron J Herrera-Estrella LR Plant biotechnology: Fast-forward genomics for improved crop production Curr Opin Plant Biol 2006 9 177 179 10.1016/j.pbi.2006.01.018

[B6] Ding Z Li S An X Liu X Qin H Wang D Transgenic expression of *MYB15 *confers enhanced sensitivity to abscisic acid and improved drought tolerance in *Arabidopsis thaliana* J Genet Genomics 2009 36 17 29 10.1016/S1673-8527(09)60003-5 19161942

[B7] Kang JY Choi HI Im MY Kim SY Arabidopsis basic leucine zipper proteins that mediate stress-responsive abscisic acid signaling Plant Cell 2002 14 343 357 10.1105/tpc.010362 11884679 PMC152917

[B8] Liu Q Kasuga M Sakuma Y Abe H Miura S Yamaguchi-Shinozaki K Shinozaki K Two transcription factors, DREB1 and DREB2, with an EREBP/AP2 DNA binding domain separate two cellular signal transduction pathways in drought- and low-temperature-responsive gene expression, respectively, in Arabidopsis Plant Cell 1998 10 1391 1406 10.1105/tpc.10.8.1391 9707537 PMC144379

[B9] Sakuma Y Maruyama K Osakabe Y Qin F Seki M Shinozaki K Yamaguchi-Shinozaki K Functional analysis of an *Arabidopsis *transcription factor, DREB2A, involved in drought-responsive gene expression Plant Cell 2006 18 1292 1309 10.1105/tpc.105.035881 16617101 PMC1456870

[B10] Hu H Dai M Yao J Xiao B Li X Zhang Q Xiong L Overexpressing a NAM, ATAF, and CUC (NAC) transcription factor enhances drought resistance and salt tolerance in rice Proc Natl Acad Sci USA 2006 103 12987 12992 10.1073/pnas.0604882103 16924117 PMC1559740

[B11] Oh SJ Song SI Kim YS Jang HJ Kim SY Kim M Kim YK Nahm BH Kim JK Arabidopsis CBF3/DREB1A and ABF3 in transgenic rice increased tolerance to abiotic stress without stunting growth Plant Physiol 2005 138 341 351 10.1104/pp.104.059147 15834008 PMC1104188

[B12] Yi K Wu Z Zhou J Du L Guo L Wu Y Wu P *OsPTF1*, a novel transcription factor involved in tolerance to phosphate starvation in rice Plant Physiol 2005 138 2087 2096 10.1104/pp.105.063115 16006597 PMC1183397

[B13] Nelson DE Repetti PP Adams TR Creelman RA Wu J Warner DC Anstrom DC Bensen RJ Castiglioni PP Donnarummo MG Plant nuclear factor Y (NF-Y) B subunits confer drought tolerance and lead to improved corn yields on water-limited acres Proc Natl Acad Sci USA 2007 104 16450 16455 10.1073/pnas.0707193104 17923671 PMC2034233

[B14] Winicov I Bastola DR Transgenic overexpression of the transcription factor *alfin1 *enhances expression of the endogenous *MsPRP2 *gene in alfalfa and improves salinity tolerance of the plants Plant Physiol 1999 120 473 480 10.1104/pp.120.2.473 10364398 PMC59285

[B15] Miki B Abdeen A Manabe Y MacDonald P Selectable marker genes and unintended changes to the plant transcriptome Plant Biotechnol J 2009 7 211 218 10.1111/j.1467-7652.2009.00400.x 19261135

[B16] Eulgem T Rushton PJ Robatzek S Somssich IE The WRKY superfamily of plant transcription factors Trends Plant Sci 2000 5 199 206 10.1016/S1360-1385(00)01600-9 10785665

[B17] Rijpkema AS Gerats T Vandenbussche M Evolutionary complexity of MADS complexes Curr Opin Plant Biol 2007 10 32 38 10.1016/j.pbi.2006.11.010 17140839

[B18] Abdeen A Miki B The pleiotropic effects of the *bar *gene and glufosinate on the *Arabidopsis *transcriptome Plant Biotechnol J 2009 7 266 282 10.1111/j.1467-7652.2008.00398.x 19222808

[B19] Baker JM Hawkins ND Ward JL Lovegrove A Napier JA Shewry PR Beale MH A metabolomic study of substantial equivalence of field-grown genetically modified wheat Plant Biotechnol J 2006 4 381 392 10.1111/j.1467-7652.2006.00197.x 17177804

[B20] Baudo MM Lyons R Powers S Pastori GM Edwards KJ Holdsworth MJ Shewry PR Transgenesis has less impact on the transcriptome of wheat grain than conventional breeding Plant Biotechnol J 2006 4 369 380 10.1111/j.1467-7652.2006.00193.x 17177803

[B21] Albo AG Mila S Digilio G Motto M Aime S Corpillo D Proteomic analysis of a genetically modified maize flour carrying CRY1AB gene and comparison to the corresponding *wild-type* Maydica 2007 52 443 455

[B22] Catchpole GS Beckmann M Enot DP Mondhe M Zywicki B Taylor J Hardy N Smith A King RD Kell DB Hierarchical metabolomics demonstrates substantial compositional similarity between genetically modified and conventional potato crops Proc Natl Acad Sci USA 2005 102 14458 14462 10.1073/pnas.0503955102 16186495 PMC1242293

[B23] Cheng KC Beaulieu J Iquira E Belzile FJ Fortin MG Strömvik MV Effect of transgenes on global gene expression in soybean is within the natural range of variation of conventional cultivars J Agric Food Chem 2008 56 3057 3067 10.1021/jf073505i 18433101

[B24] Corpillo D Gardini G Vaira AM Basso M Aime S Accotto GP Fasano M Proteomics as a tool to improve investigation of substantial equivalence in genetically modified organisms: The case of a virus-resistant tomato Proteomics 2004 4 193 200 10.1002/pmic.200300540 14730681

[B25] El Ouakfaoui S Miki B The stability of the Arabidopsis transcriptome in transgenic plants expressing the marker genes *nptII *and *uidA* Plant J 2005 41 791 800 10.1111/j.1365-313X.2005.02350.x 15743445

[B26] Gregersen PL Brinch-Pedersen H Holm PB A microarray-based comparative analysis of gene expression profiles during grain development in transgenic and wild type wheat Transgenic Res 2005 14 887 905 10.1007/s11248-005-1526-y 16315094

[B27] Lehesranta SJ Davies HV Shepherd LVT Nunan N McNicol JW Auriola S Koistinen KM Suomalainen S Kokko HI Kärenlampi SO Comparison of tuber proteomes of potato varieties, landraces, and genetically modified lines Plant Physiol 2005 138 1690 1699 10.1104/pp.105.060152 15951487 PMC1176438

[B28] Jakoby M Weisshaar B Dröge-Laser W Vicente-Carbajosa J Tiedemann J Kroj T Parcy F bZIP transcription factors in *Arabidopsis* Trends Plant Sci 2002 7 106 111 10.1016/S1360-1385(01)02223-3 11906833

[B29] Choi H Hong J Ha J Kang J Kim SY ABFs, a family of ABA-responsive element binding factors J Biol Chem 2000 275 1723 1730 10.1074/jbc.275.3.1723 10636868

[B30] Shinozaki K Yamaguchi-Shinozaki K Gene networks involved in drought stress response and tolerance J Exp Bot 2007 58 221 227 10.1093/jxb/erl164 17075077

[B31] Uno Y Furihata T Abe H Yoshida R Shinozaki K Yamaguchi-Shinozaki K Arabidopsis basic leucine zipper transcription factors involved in an abscisic acid-dependent signal transduction pathway under drought and high-salinity conditions Proc Natl Acad Sci USA 2000 97 11632 11637 10.1073/pnas.190309197 11005831 PMC17252

[B32] Kim JB Kang JY Kim SY Over-expression of a transcription factor regulating ABA-responsive gene expression confers multiple stress tolerance Plant Biotechnol J 2004 2 459 466 10.1111/j.1467-7652.2004.00090.x 17168892

[B33] Kim S Kang JY Cho DI Park JH Kim SY ABF2, an ABRE-binding bZIP factor, is an essential component of glucose signaling and its overexpression affects multiple stress tolerance Plant J 2004 40 75 87 10.1111/j.1365-313X.2004.02192.x 15361142

[B34] Seki M Narusaka M Ishida J Nanjo T Fujita M Oono Y Kamiya A Nakajima M Enju A Sakurai T Monitoring the expression profiles of 7000 *Arabidopsis *genes under drought, cold and high-salinity stresses using a full-length cDNA microarray Plant J 2002 31 279 292 10.1046/j.1365-313X.2002.01359.x 12164808

[B35] Seki M Narusaka M Abe H Kasuga M Yamaguchi-Shinozaki K Carninci P Hayashizaki Y Shinozaki K Monitoring the expression pattern of 1300 Arabidopsis genes under drought and cold stresses by using a full-length cDNA microarray Plant Cell 2001 13 61 72 10.1105/tpc.13.1.61 11158529 PMC102214

[B36] Matsui A Ishida J Morosawa T Mochizuki Y Kaminuma E Endo TA Okamoto M Nambara E Nakajima M Kawashima M *Arabidopsis *transcriptome analysis under drought, cold, high-salinity and ABA treatment conditions using a tiling array Plant Cell Physiol 2008 49 1135 1149 10.1093/pcp/pcn101 18625610

[B37] Nakashima K Shinwari ZK Sakuma Y Seki M Miura S Shinozaki K Yamaguchi-Shinozaki K Organization and expression of two *Arabidopsis DREB2 *genes encoding DRE-binding proteins involved in dehydration- and high-salinity-responsive gene expression Plant Mol Biol 2000 42 657 665 10.1023/A:1006321900483 10809011

[B38] Haake V Cook D Riechmann JL Pineda O Thomashow MF Zhang JZ Transcription factor CBF4 is a regulator of drought adaptation in Arabidopsis Plant Physiol 2002 130 639 648 10.1104/pp.006478 12376631 PMC166593

[B39] Abe H Urao T Ito T Seki M Shinozaki K Yamaguchi-Shinozaki K Arabidopsis AtMYC2 (bHLH) and AtMYB2 (MYB) function as transcriptional activators in abscisic acid signaling Plant Cell 2003 15 63 78 10.1105/tpc.006130 12509522 PMC143451

[B40] Abe H Yamaguchi-Shinozaki K Urao T Iwasaki T Hosokawa D Shinozaki K Role of arabidopsis MYC and MYB homologs in drought- and abscisic acid-regulated gene expression Plant Cell 1997 9 1859 1868 10.1105/tpc.9.10.1859 9368419 PMC157027

[B41] Urao T Yamaguchi-Shinozaki K Urao S Shinozaki K An Arabidopsis *myb *homolog is induced by dehydration stress and its gene product binds to the conserved MYB recognition sequence Plant Cell 1993 5 1529 1539 10.1105/tpc.5.11.1529 8312738 PMC160383

[B42] Tran LSP Nakashima K Sakuma Y Simpson SD Fujita Y Maruyama K Fujita M Seki M Shinozaki K Yamaguchi-Shinozaki K Isolation and functional analysis of Arabidopsis stress-inducible NAC transcription factors that bind to a drought-responsive *cis*-element in the *early responsive to dehydration stress 1 *promoter Plant Cell 2004 16 2481 2498 10.1105/tpc.104.022699 15319476 PMC520947

[B43] Tran LSP Nakashima K Sakuma Y Osakabe Y Qin F Simpson SD Maruyama K Fujita Y Shinozaki K Yamaguchi-Shinozaki K Co-expression of the stress-inducible zinc finger homeodomain ZFHD1 and NAC transcription factors enhances expression of the *ERD1 *gene in Arabidopsis Plant J 2006 49 46 63 10.1111/j.1365-313X.2006.02932.x 17233795

[B44] Gilbertson L Cre-*lox *recombination: Cre-ative tools for plant biotechnology Trends Biotechnol 2003 21 550 555 10.1016/j.tibtech.2003.09.011 14624864

[B45] De Buck S Peck I De Wilde C Marjanac G Nolf J De Paepe A Depicker A Generation of single-copy T-DNA transformants in Arabidopsis by the CRE/*loxP *recombination-mediated resolution system Plant Physiol 2007 145 1171 1182 10.1104/pp.107.104067 17693537 PMC2151725

[B46] Hoa TTC Bong BB Huq E Hodges TK Cre/*lox *site-specific recombination controls the excision of a transgene from the rice genome Theor Appl Genet 2002 104 518 525 10.1007/s001220100748 12582653

[B47] Osborne BI Wirtz U Baker B A system for insertional mutagenesis and chromosomal rearrangement using the Ds transposon and Cre-*lox* Plant J 1995 7 687 701 10.1046/j.1365-313X.1995.7040687.x 7742862

[B48] Russell SH Hoopes JL Odell JT Directed excision of a transgene from the plant genome Mol Genet Genet 1992 234 49 59 10.1007/BF00272344 1495484

[B49] Coppoolse ER de Vroomen MJ Roelofs D Smit J van Gennip F Hersmus BJM Nijkamp HJJ van Haaren MJJ Cre recombinase expression can result in phenotypic aberrations in plants Plant Mol Biol 2003 51 263 279 10.1023/A:1021174726070 12602884

[B50] Que Q Wang HY Jorgensen RA Distinct patterns of pigment suppression are produced by allelic sense and antisense chalcone synthase transgenes in petunia flowers Plant J 1998 13 401 409 10.1046/j.1365-313X.1998.00038.x

[B51] Corneille S Lutz K Svab Z Maliga P Efficient elimination of selectable marker genes from the plastid genome by the CRE-*lox *site-specific recombination system Plant J 2001 27 171 178 10.1046/j.1365-313x.2001.01068.x 11489194

[B52] Corneille S Lutz KA Azhagiri AK Maliga P Identification of functional *lox *sites in the plastid genome Plant J 2003 35 753 762 10.1046/j.1365-313X.2003.01845.x 12969428

[B53] Hajdukiewicz PTJ Gilbertson L Staub JM Multiple pathways for Cre/*lox*-mediated recombination in plastids Plant J 2001 27 161 170 10.1046/j.1365-313x.2001.01067.x 11489193

[B54] Heidmann D Lehner CF Reduction of Cre recombinase toxicity in proliferating *Drosophila *cells by estrogen-dependent activity regulation Dev Genes Evol 2001 211 458 465 10.1007/s004270100167 11685583

[B55] Loonstra A Vooijs M Beverloo HB Allak BA van Drunen E Kanaar R Berns A Jonkers J Growth inhibition and DNA damage induced by Cre recombinase in mammalian cells Proc Natl Acad Sci USA 2001 98 9209 9214 10.1073/pnas.161269798 11481484 PMC55399

[B56] Schmidt EE Taylor DS Prigge JR Barnett S Capecchi MR Illegitimate Cre-dependent chromosome rearrangements in transgenic mouse spermatids Proc Natl Acad Sci USA 2000 97 13702 13707 10.1073/pnas.240471297 11087830 PMC17639

[B57] Silver DP Livingston DM Self-excising retroviral vectors encoding the Cre recombinase overcome Cre-mediated cellular toxicity Mol Cell 2001 8 233 243 10.1016/S1097-2765(01)00295-7 11511376

[B58] Li J Brader G Palva ET The WRKY70 Transcription Factor: A Node of Convergence for Jasmonate-Mediated and Salicylate-Mediated Signals in Plant Defense Plant Cell 2004 16 319 331 10.1105/tpc.016980 14742872 PMC341906

[B59] Agarwal PK Agarwal P Reddy MK Sopory SK Role of DREB transcription factors in abiotic and biotic stress tolerance in plants Plant Cell Rep 2006 25 1263 1274 10.1007/s00299-006-0204-8 16858552

[B60] Kasuga M Liu Q Miura S Yamaguchi-Shinozaki K Shinozaki K Improving plant drought, salt, and freezing tolerance by gene transfer of a single stress-inducible transcription factor Nat Biotechnol 1999 17 287 291 10.1038/7036 10096298

[B61] Novillo F Alonso JM Ecker JR Salinas J CBF2/DREB1C is a negative regulator of *CBF1/DREB1B *and *CBF3/DREB1A *expression and plays a central role in stress tolerance in *Arabidopsis* Proc Natl Acad Sci USA 2004 101 3985 3990 10.1073/pnas.0303029101 15004278 PMC374356

[B62] Gómez-Porras JL Riaño-Pachón DM Dreyer I Mayer JE Mueller-Roeber B Genome-wide analysis of ABA-responsive elements ABRE and CE3 reveals divergent patterns in Arabidopsis and rice BMC Genomics 2007 8 260 10.1186/1471-2164-8-260 17672917 PMC2000901

[B63] Hugouvieux V Kwak JM Schroeder JI An mRNA cap binding protein, ABH1, modulates early abscisic acid signal transduction in *Arabidopsis* Cell 2001 106 477 487 10.1016/S0092-8674(01)00460-3 11525733

[B64] Lu C Fedoroff N A mutation in the Arabidopsis *HYL1 *gene encoding a dsRNA binding protein affects responses to abscisic acid, auxin, and cytokinin Plant Cell 2000 12 2351 2365 10.1105/tpc.12.12.2351 11148283 PMC102223

[B65] Nishimura N Kitahata N Seki M Narusaka Y Narusaka M Kuromori T Asami T Shinozaki K Hirayama T Analysis of *ABA hypersensitive germination2 *revealed the pivotal functions of PARN in stress response in Arabidopsis Plant J 2005 44 972 984 10.1111/j.1365-313X.2005.02589.x 16359390

[B66] Papp I Mur LA Dalmadi A Dulai S Koncz C A mutation in the *Cap Binding Protein 20 *gene confers drought tolerance to *Arabidopsis* Plant Mol Biol 2004 55 679 686 10.1007/s11103-004-1680-2 15604709

[B67] Rumeau D Peltier G Cournac L Chlororespiration and cyclic electron flow around PSI during photosynthesis and plant stress response Plant Cell Environ 2007 30 1041 1051 10.1111/j.1365-3040.2007.01675.x 17661746

[B68] Casano LM Zapata JM Martín M Sabater B Chlororespiration and poising of cyclic electron transport. Plastoquinone as electron transporter between thylakoid NADH dehydrogenase and peroxidase J Biol Chem 2000 275 942 948 10.1074/jbc.275.2.942 10625631

[B69] Grandbastien M-A Activation of plant retrotransposons under stress conditions Trends Plant Sci 1998 3 181 187 10.1016/S1360-1385(98)01232-1

[B70] Madlung A Comai L The effect of stress on genome regulation and structure Ann Bot 2004 94 481 495 10.1093/aob/mch172 15319229 PMC4242226

[B71] Sauer B Identification of cryptic lox sites in the yeast genome by selection for Cre-mediated chromosome translocations that confer multiple drug resistance J Mol Biol 1992 223 911 928 10.1016/0022-2836(92)90252-F 1554399

[B72] Thyagarajan B Guimarães MJ Groth AC Calos MP Mammalian genomes contain active recombinase recognition sites Gene 2000 244 47 54 10.1016/S0378-1119(00)00008-1 10689186

[B73] Drescher A Ruf S Calsa T Jr Carrer H Bock R The two largest chloroplast genome-encoded open reading frames of higher plants are essential genes Plant J 2000 22 97 104 10.1046/j.1365-313x.2000.00722.x 10792825

[B74] Shikanai T Shimizu K Ueda K Nishimura Y Kuroiwa T Hashimoto T The chloroplast *clpP *gene, encoding a proteolytic subunit of ATP-dependent protease, is indispensable for chloroplast development in tobacco Plant Cell Physiol 2001 42 264 273 10.1093/pcp/pce031 11266577

[B75] Legen J Wanner G Herrmann RG Small I Schmitz-Linneweber C Plastid tRNA genes *trnC-GCA *and *trnN-GUU *are essential for plant cell development Plant J 2007 51 751 762 10.1111/j.1365-313X.2007.03177.x 17573798

[B76] Bensmihen S Giraudat J Parcy F Characterization of three homologous basic leucine zipper transcription factors (bZIP) of the ABI5 family during *Arabidopsis thaliana *embryo maturation J Exp Bot 2005 56 597 603 10.1093/jxb/eri050 15642716

[B77] Fujita Y Fujita M Satoh R Maruyama K Parvez MM Seki M Hiratsu K Ohme-Takagi M Shinozaki K Yamaguchi-Shinozaki K AREB1 is a transcription activator of novel ABRE-dependent ABA signaling that enhances drought stress tolerance in *Arabidopsis* Plant Cell 2005 17 3470 3488 10.1105/tpc.105.035659 16284313 PMC1315382

[B78] Lopez-Molina L Mongrand S Chua NH A postgermination developmental arrest checkpoint is mediated by abscisic acid and requires the ABI5 transcription factor in *Arabidopsis* Proc Natl Acad Sci USA 2001 98 4782 4787 10.1073/pnas.081594298 11287670 PMC31911

[B79] Chae MJ Lee JS Nam MH Cho K Hong JY Yi SA Suh SC Yoon IS A rice dehydration-inducible SNF1-related protein kinase 2 phosphorylates an abscisic acid responsive element-binding factor and associates with ABA signaling Plant Mol Biol 2007 63 151 169 10.1007/s11103-006-9079-x 16977424

[B80] Fujii H Verslues PE Zhu JK Identification of two protein kinases required for abscisic acid regulation of seed germination, root growth, and gene expression in *Arabidopsis* Plant Cell 2007 19 485 494 10.1105/tpc.106.048538 17307925 PMC1867333

[B81] Furihata T Maruyama K Fujita Y Umezawa T Yoshida R Shinozaki K Yamaguchi-Shinozaki K Abscisic acid-dependent multisite phosphorylation regulates the activity of a transcription activator AREB1 Proc Natl Acad Sci USA 2006 103 1988 1993 10.1073/pnas.0505667103 16446457 PMC1413621

[B82] Johnson RR Wagner RL Verhey SD Walker-Simmons MK The abscisic acid-responsive kinase PKABA1 interacts with a seed-specific abscisic acid response element-binding factor, TaABF, and phosphorylates TaABF peptide sequences Plant Physiol 2002 130 837 846 10.1104/pp.001354 12376648 PMC166610

[B83] Kagaya Y Hobo T Murata M Ban A Hattori T Abscisic acid-induced transcription is mediated by phosphorylation of an abscisic acid response element binding factor, TRAB1 Plant Cell 2002 14 3177 3189 10.1105/tpc.005272 12468735 PMC151210

[B84] Kobayashi Y Murata M Minami H Yamamoto S Kagaya Y Hobo T Yamamoto A Hattori T Abscisic acid-activated SNRK2 protein kinases function in the gene-regulation pathway of ABA signal transduction by phosphorylating ABA response element-binding factors Plant J 2005 44 939 949 10.1111/j.1365-313X.2005.02583.x 16359387

[B85] Choi H Park HJ Park JH Kim S Im MY Seo HH Kim YW Hwang I Kim SY Arabidopsis calcium-dependent protein kinase AtCPK32 interacts with ABF4, a transcriptional regulator of abscisic acid-responsive gene expression, and modulates its activity Plant Physiol 2005 139 1750 1761 10.1104/pp.105.069757 16299177 PMC1310556

[B86] Zhu SY Yu XC Wang XJ Zhao R Li Y Fan RC Shang Y Du SY Wang XF Wu FQ Two calcium-dependent protein kinases, CPK4 and CPK11, regulate abscisic acid signal transduction in Arabidopsis Plant Cell 2007 19 3019 3036 10.1105/tpc.107.050666 17921317 PMC2174700

[B87] Kim S Choi H Ryu HJ Park JH Kim MD Kim SY ARIA, an Arabidopsis arm repeat protein interacting with a transcriptional regulator of abscisic acid-responsive gene expression, is a novel abscisic acid signaling component Plant Physiol 2004 136 3639 3648 10.1104/pp.104.049189 15516505 PMC527162

[B88] Finkelstein R Gampala SSL Lynch TJ Thomas TL Rock CD Redundant and distinct functions of the ABA response loci *ABA-INSENSITIVE(ABI)5 *and *ABRE-BINDING FACTOR (ABF)3* Plant Mol Biol 2005 59 253 267 10.1007/s11103-005-8767-2 16247556

[B89] Nakamura S Lynch TJ Finkelstein RR Physical interactions between ABA response loci of *Arabidopsis* Plant J 2001 26 627 635 10.1046/j.1365-313x.2001.01069.x 11489176

[B90] Narusaka Y Nakashima K Shinwari ZK Sakuma Y Furihata T Abe H Narusaka M Shinozaki K Yamaguchi-Shinozaki K Interaction between two *cis*-acting elements, ABRE and DRE, in ABA-dependent expression of *Arabidopsis rd29A *gene in response to dehydration and high-salinity stresses Plant J 2003 34 137 148 10.1046/j.1365-313X.2003.01708.x 12694590

[B91] Kim SY Ma J Perret P Li Z Thomas TL Arabidopsis ABI5 subfamily members have distinct DNA-binding and transcriptional activities Plant Physiol 2002 130 688 697 10.1104/pp.003566 12376636 PMC166598

[B92] Yang Y Costa A Leonhardt N Siegel RS Schroeder JI Isolation of a strong *Arabidopsis *guard cell promoter and its potential as a research tool Plant Methods 2008 4 6 10.1186/1746-4811-4-6 18284694 PMC2323621

[B93] Kuhn JM Schroeder JI Impacts of altered RNA metabolism on abscisic acid signaling Curr Opin Plant Biol 2003 6 463 469 10.1016/S1369-5266(03)00084-0 12972047

[B94] Lee B Kapoor A Zhu J Zhu JK STABILIZED1, a stress-upregulated nuclear protein, is required for pre-mRNA splicing, mRNA turnover, and stress tolerance in Arabidopsis Plant Cell 2006 18 1736 1749 10.1105/tpc.106.042184 16751345 PMC1488911

[B95] Xiong L Gong Z Rock CD Subramanian S Guo Y Xu W Galbraith D Zhu JK Modulation of abscisic acid signal transduction and biosynthesis by an Sm-like protein in *Arabidopsis* Dev Cell 2001 1 771 781 10.1016/S1534-5807(01)00087-9 11740939

[B96] Koiwa H Barb AW Xiong L Li F McCully MG Lee BH Sokolchik I Zhu J Gong Z Reddy M C-terminal domain phosphatase-like family members (AtCPLs) differentially regulate *Arabidopsis thaliana *abiotic stress signaling, growth, and development Proc Natl Acad Sci USA 2002 99 10893 10898 10.1073/pnas.112276199 12149434 PMC125069

[B97] Xiong L Lee H Ishitani M Tanaka Y Stevenson B Koiwa H Bressan RA Hasegawa PM Zhu JK Repression of stress-responsive genes by FIERY2, a novel transcriptional regulator in *Arabidopsis* Proc Natl Acad Sci USA 2002 99 10899 10904 10.1073/pnas.162111599 12149453 PMC125070

[B98] Perera IY Hung CY Moore CD Stevenson-Paulik J Boss WF Transgenic Arabidopsis plants expressing the type 1 inositol 5-phosphatase exhibit increased drought tolerance and altered abscisic acid signaling Plant Cell 2008 20 2876 2893 10.1105/tpc.108.061374 18849493 PMC2590728

[B99] Atkin OK Macherel D The crucial role of plant mitochondria in orchestrating drought tolerance Ann Bot 2009 103 581 597 10.1093/aob/mcn094 18552366 PMC2707344

[B100] Chaves MM Flexas J Pinheiro C Photosynthesis under drought and salt stress: regulation mechanisms from whole plant to cell Ann Bot 2009 103 551 560 10.1093/aob/mcn125 18662937 PMC2707345

[B101] Carvalho IS Chaves MM Pinto Ricardo C Influence of Water Stress on the Chemical Composition of Seeds of Two Lupins (*Lupinus albus *and *Lupinus mutabilis*) J Agron Crop Sci 2005 191 95 98 10.1111/j.1439-037X.2004.00128.x

[B102] Champolivier L Merrien A Effects of water stress applied at different growth stages to *Brassica napus *L. var. *oleifera *on yield, yield components and seed quality Eur J Agron 1996 5 153 160 10.1016/S1161-0301(96)02004-7

[B103] Dornbos DL Jr Mullen RE Soybean seed protein and oil contents and fatty acid composition adjustments by drought and temperature J Am Oil Chem Soc 1992 69 228 231 10.1007/BF02635891

[B104] Gigon A Matos AR Laffray D Zuily-Fodil Y Pham-Thi AT Effect of drought stress on lipid metabolism in the leaves of *Arabidopsis thaliana *(ecotype Columbia) Ann Bot 2004 94 345 351 10.1093/aob/mch150 15277243 PMC4242175

[B105] Jensen CR Mogensen VO Mortensen G Fieldsend JK Milford GFJ Andersen MN Thage JH Seed glucosinolate, oil and protein contents of field-grown rape (*Brassica napus *L.) affected by soil drying and evaporative demand Field Crops Res 1996 47 93 105 10.1016/0378-4290(96)00026-3

[B106] Rotundo JL Westgate ME Meta-analysis of environmental effects on soybean seed composition Field Crops Res 2009 110 147 156 10.1016/j.fcr.2008.07.012

[B107] Dakhma WS Zarrouk M Cherif A Effects of drought-stress on lipids in rape leaves Phytochemistry 1995 40 1383 1386 10.1016/0031-9422(95)00459-K

[B108] Bejarano L Mignolet E Devaux A Espinola N Carrasco E Larondelle Y Glycoalkaloids in potato tubers: the effect of variety and drought stress on the α-solanine and α-chaconine contents of potatoes J Sci Food Agric 2000 80 2096 2100 10.1002/1097-0010(200011)80:14<2096::AID-JSFA757>3.0.CO;2-6

[B109] Finkelstein RR Lynch TJ The Arabidopsis abscisic acid response gene *ABI5 *encodes a basic leucine zipper transcription factor Plant Cell 2000 12 599 610 10.1105/tpc.12.4.599 10760247 PMC139856

[B110] Lopez-Molina L Mongrand S McLachlin DT Chait BT Chua NH ABI5 acts downstream of ABI3 to execute an ABA-dependent growth arrest during germination Plant J 2002 32 317 328 10.1046/j.1365-313X.2002.01430.x 12410810

[B111] Carles C Bies-Etheve N Aspart L Léon-Kloosterziel KM Koornneef M Echeverria M Delseny M Regulation of *Arabidopsis thaliana Em *genes: role of ABI5 The Plant Journal 2002 30 373 383 10.1046/j.1365-313X.2002.01295.x 12000684

[B112] Zimmermann P Hirsch-Hoffmann M Hennig L Gruissem W GENEVESTIGATOR. Arabidopsis microarray database and analysis toolbox Plant Physiol 2004 136 2621 2632 10.1104/pp.104.046367 15375207 PMC523327

[B113] Nakashima K Fujita Y Katsura K Maruyama K Narusaka Y Seki M Shinozaki K Yamaguchi-Shinozaki K Transcriptional regulation of ABI3- and ABA-responsive genes including *RD29B *and *RD29A *in seeds, germinating embryos, and seedlings of *Arabidopsis* Plant Mol Biol 2006 60 51 68 10.1007/s11103-005-2418-5 16463099

[B114] Brocard IM Lynch TJ Finkelstein RR Regulation and role of the Arabidopsis *abscisic acid-insensitive 5 *gene in abscisic acid, sugar, and stress response Plant Physiol 2002 129 1533 1543 10.1104/pp.005793 12177466 PMC166741

[B115] Clough SJ Bent AF Floral dip: a simplified method for *Agrobacterium*-mediated transformation of *Arabidopsis thaliana* Plant J 1998 16 735 743 10.1046/j.1365-313x.1998.00343.x 10069079

[B116] Irizarry RA Hobbs B Collin F Beazer-Barclay YD Antonellis KJ Scherf U Speed TP Exploration, normalization, and summaries of high density oligonucleotide array probe level data Biostatistics 2003 4 249 264 10.1093/biostatistics/4.2.249 12925520

[B117] Gentleman RC Carey VJ Bates DM Bolstad B Dettling M Dudoit S Ellis B Gautier L Ge Y Gentry J Bioconductor: open software development for computational biology and bioinformatics Genome Biol 2004 5 R80 10.1186/gb-2004-5-10-r80 15461798 PMC545600

[B118] Smyth GK Linear models and empirical Bayes methods for assessing differential expression in microarray experiments Stat Appl Genet Mol Biol 2004 3 Article 3 16646809 10.2202/1544-6115.1027

