# Supplementary figures and images for: Transcriptome analysis reveals absence of unintended effects in drought-tolerant transgenic plants overexpressing the transcription factor ABF3
Source: BMC Genomics. 2010 Jan 28;11:69. doi: 10.1186/1471-2164-11-69 (PMC2837038; doi:10.1186/1471-2164-11-69)

A

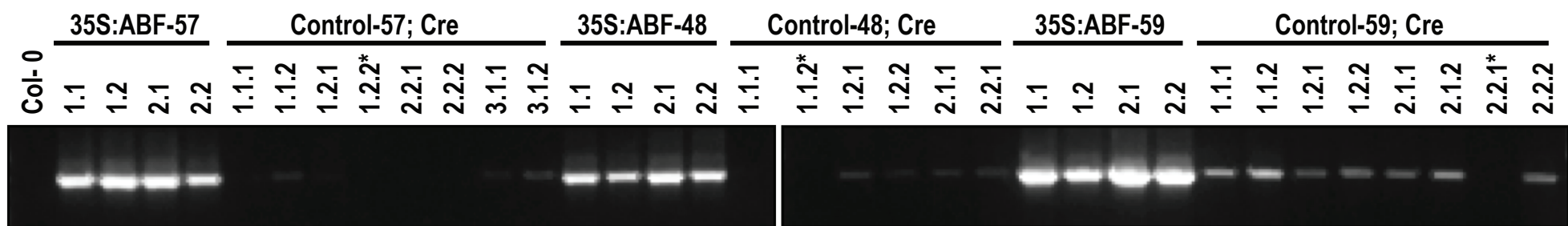

B

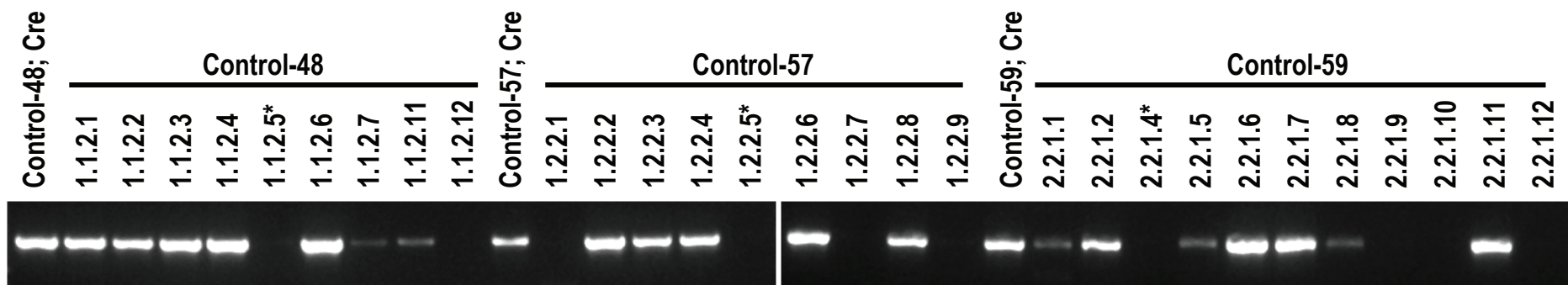

Supplement: Additional file 1 — PCR genotyping of control plant lines. (A) Control plant lines, derived from 35S:ABF3 plants crossed with plants expressing Cre recombinase, were PCR genotyped to identify plants from which the 35S-ABF3-nos transgene was excised. (B) Control plant lines following a backcross with Col-0 plants were genotyped to identify plants that lost the Cre gene by genetic segregation. Plant lines with the desired genotype that were selected following each cross are indicated with asterisks. [file 1471-2164-11-69-S1.PDF]

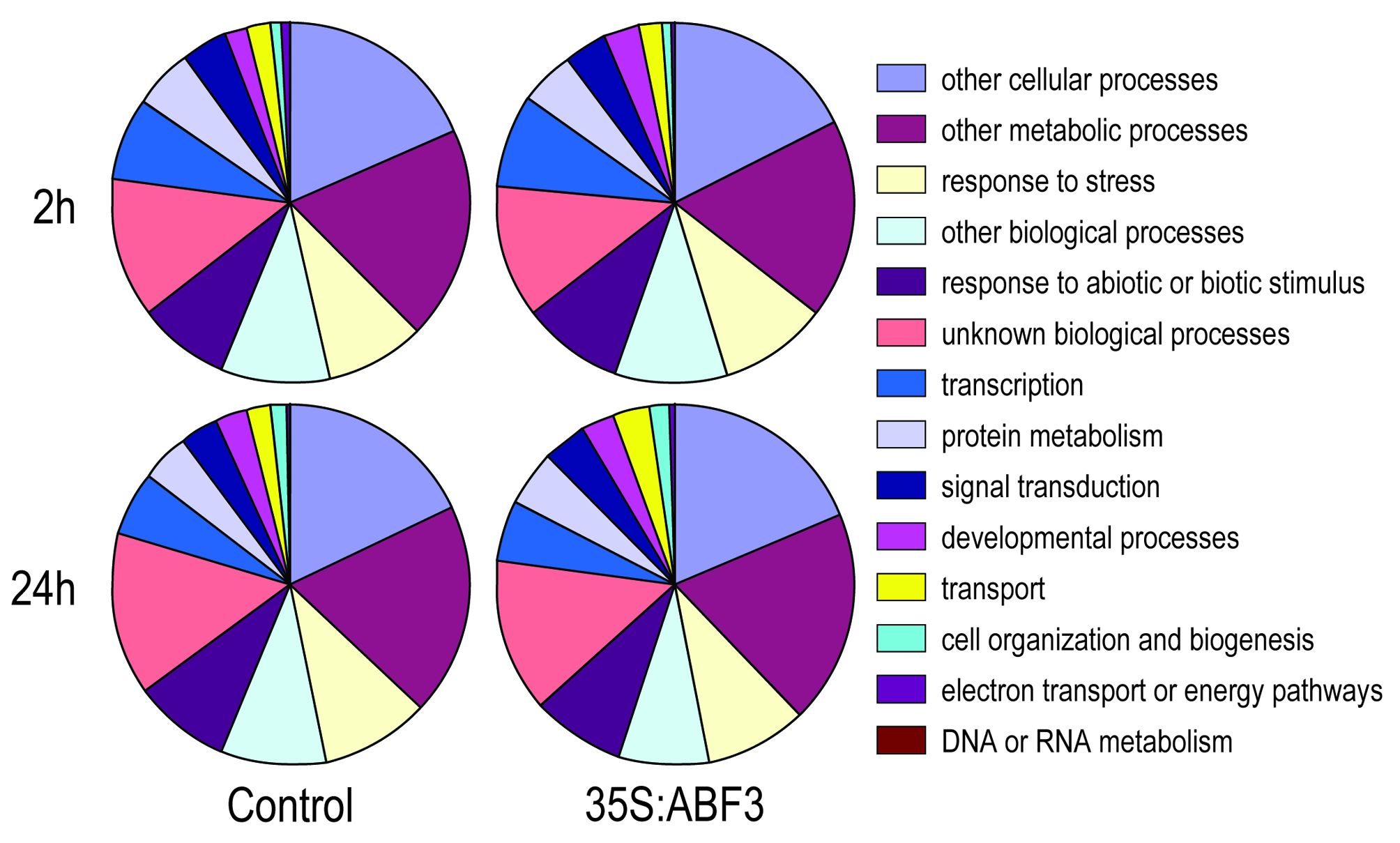

Supplement: Additional file 2 — Functional categorization of drought responsive genes in 35S:ABF3 and control plants. Distribution of genes differentially expressed in 35S:ABF3 and control plant lines at 2 h and 24 h following drought stress into functional categories. [file 1471-2164-11-69-S2.TIFF]
